# Automated tracking and quantification of angiogenic vessel formation in 3D microfluidic devices

**DOI:** 10.1371/journal.pone.0186465

**Published:** 2017-11-14

**Authors:** Mengmeng Wang, Lee-Ling Sharon Ong, Justin Dauwels, H. Harry Asada

**Affiliations:** 1 School of Electrical and Electronic Engineering, Nanyang Technological University (NTU), Singapore, Singapore; 2 Singapore-MIT Alliance for Research and Technology, Singapore, Singapore; 3 Department of Mechanical Engineering, Massachusetts Institute of Technology (MIT), Cambridge, Massachusetts, United States of America; Pennsylvania State Hershey College of Medicine, UNITED STATES

## Abstract

Angiogenesis, the growth of new blood vessels from pre-existing vessels, is a critical step in cancer invasion. Better understanding of the angiogenic mechanisms is required to develop effective antiangiogenic therapies for cancer treatment. We culture angiogenic vessels in 3D microfluidic devices under different Sphingosin-1-phosphate (S1P) conditions and develop an automated vessel formation tracking system (AVFTS) to track the angiogenic vessel formation and extract quantitative vessel information from the experimental time-lapse phase contrast images. The proposed AVFTS first preprocesses the experimental images, then applies a distance transform and an augmented fast marching method in skeletonization, and finally implements the Hungarian method in branch tracking. When applying the AVFTS to our experimental data, we achieve 97.3% precision and 93.9% recall by comparing with the ground truth obtained from manual tracking by visual inspection. This system enables biologists to quantitatively compare the influence of different growth factors. Specifically, we conclude that the positive S1P gradient increases cell migration and vessel elongation, leading to a higher probability for branching to occur. The AVFTS is also applicable to distinguish tip and stalk cells by considering the relative cell locations in a branch. Moreover, we generate a novel type of cell lineage plot, which not only provides cell migration and proliferation histories but also demonstrates cell phenotypic changes and branch information.

## Introduction

Angiogenesis is the growth of blood vessels from pre-existing vessels, which is a highly dynamic process involving interactions between endothelial cells (ECs) and their environments [1–3]. It is a critical process in growth, development, as well as cancer invasion. During angiogenesis, ECs migrate in a coordinated manner, specializing into two distinct phenotypes: tip cells and stalk cells [4]. Tip cells sense and respond to the guidance cues through filopodia, burrow into the extracellular matrix (ECM), and form conduits. Stalk cells trail behind the tip cells along the conduits and form solid sprouts or lumen vessels. The balance between tip and stalk cell phenotypes must be tightly controlled to ensure a correct development of the vasculature [5]. Both cell phenotypes can dynamically switch positions and functions during the sprouting process [6, 7]. The inter-transition of the cell phenotypes plays an important role for ECs sprouting out from monolayer, extending and creating new branches, as well as reconnecting in a later stage [8–10].

Over many years, most of the angiogenic experiments have been performed in *in vivo* conditions. Although it provides the right environments, it is difficult to interpret the observations due to the complex nature. Alternatively, the traditional 2D *in vitro* cell culture is a simpler system, but significantly lacks many essential conditions compared to the actual *in vivo* conditions. The 3D microfluidic platform provides an environment that more closely represents the *in vivo* setup with tight control of various growth factors delivery [11, 12]. Human microvascular endothelial cells (HMVECs) are more angiogenic-like compared to human umbilical vein endothelial cells (HUVECs) as HMVECs are microvascular cells. However, existing angiogenic studies mainly use HUVECs, since HMVECs are more fastidious in the culture environments which makes long-term culture more challenging [13]. Sphingosine-1-phosphate (S1P) has been shown to increase length of the sprouts when culturing HUVECs in *in vitro* angiogenic experiments [14, 15], but there are few studies which cultured and observed angiogenic vessels with branching using HMVECs in 3D *in vitro* experiments. In order to observe angiogenic vessels with branching and investigate the influence of S1P on the HMVECs in angiogenesis, we culture HMVECs in 3D microfluidic devices (MFDs) under different S1P conditions. We acquire time-lapse phase contrast images to investigate the dynamic angiogenic vessel formation in 3D MFDs. Time-lapse imaging is a valuable tool for studying cell behaviors [16, 17]. It yields data of finer resolution than traditional “still-shot” studies and allows direct examination of cell dynamics [18].

Nowadays, automated image analysis has become a powerful tool for probing a wide variety of biological questions using microscopy [19]. Most of the existing automated image analysis systems for angiogenesis study are developed for tracking the migration of individual cells [20–26]. Only a few systems exist to quantitatively analyze the angiogenic networks in 2D *in vitro* systems, providing geometric parameters such as the average length, the number of branches, and the number of nodes [27–31]. However, quantitative analysis tools to track the angiogenic vessel formation in 3D *in vitro* cell culture systems, which would benefit for the investigation of the vessel morphological change and cell phenotypic change over time [32], has been seldom addressed. In addition, the dynamic selection and continued competition of cell phenotypes (tip and stalk cells), leading to branching and reconnection, is still not fully understood [33]. Identification of cell phenotypes from experimental images would be useful for unraveling the biological functions of different cell phenotypes and developing computational models.

Therefore, the objective of this paper is to develop an automated image system to track angiogenic vessel formation, to extract the geometric parameters such as the average vessel length, width, and the number of branches, and to identify cell phenotypes (tip/stalk cells) from the time-lapse experimental phase contrast images.

Quantitative angiogenic vessel parameters are commonly estimated from the vessel skeleton, which provides a simple and compact representation of the angiogenic vessel. Common methods for the extraction of the skeleton, namely skeletonization, are divided into two classes: morphological thinning and distance coding [34]. Morphological thinning removes the outer layers of pixels from the vascular networks successively while maintaining connectivity [35, 36]. This methodology has been implemented in [29, 30], and [31] to quantify the EC networks in 2D *in vitro* cell culture systems. However, it is time-consuming and very sensitive to the defects in the boundaries. The distance coding approach extracts skeletal points directly from the image based on the distance transform (DT) [37–41]. The skeleton lies along the singularities in the DT [42, 43]. However, the computation of the singularities of the DT is a numerically unstable and delicate process. We apply an augmented fast marching method (AFMM) to represent the vessel skeletons with connected segments and nodes [41]. A binary support vector machine (SVM) classifier is then trained to remove specious segments. Moreover, we track the trajectory of each branch by the Hungarian method. We achieve 97.3% precision and 93.9% recall when applying the proposed system to our experimental data. By considering the cell trajectories information in [26], we identify cell phenotypes and generate a novel type of cell lineage plot to demonstrate both cell and branch information.

The rest of this paper is structured as follows. In Section II, we elaborate on the procedures of the angiogenic experiments. In Section III, we explain the proposed automated vessel formation tracking system (AVFTS). In Section IV, we discuss how we apply the AVFTS to our experimental phase contrast images, present results for this analysis. Specifically, we identify cell phenotypes using AVFTS, and based on results from the AVFTS analysis, we compare the influence of S1P on angiogenic vessel morphologies and introduce a novel type of cell lineage plot. In Section V, we offer concluding remarks.

## Experimental methods

**Fig 1:**
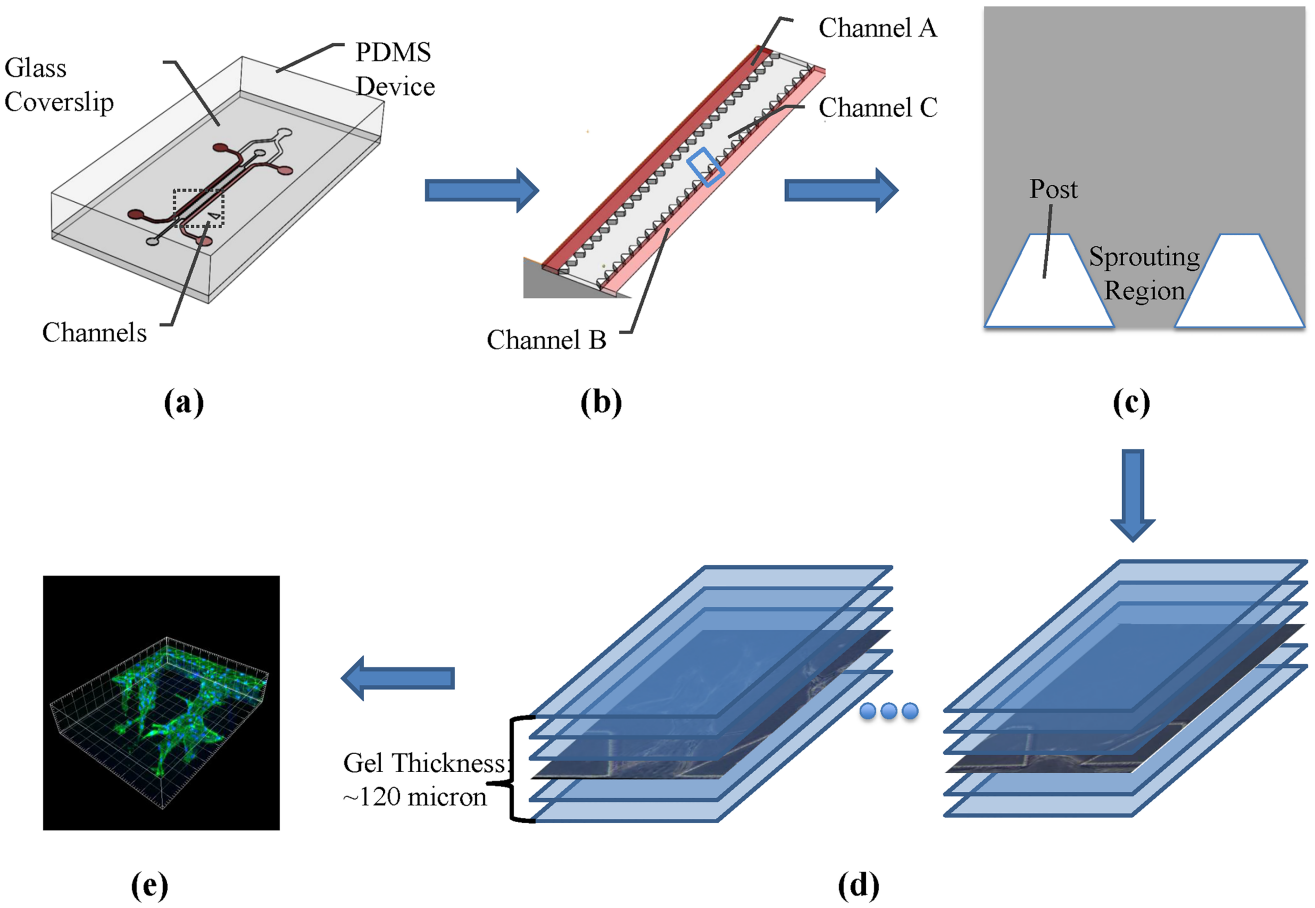
Experiment and image acquisition procedure. (a) External appearance of MFD. (b) Internal appearance of MFD with 37 sprouting regions. (c) An individual sprouting region separated by posts. (d) Time-lapse phase contrast images with one-day interval. (e) End-point confocal image.

Our angiogenic experiments are conducted in the MFDs, as illustrated in Fig 1(a) [11]. MFDs, with channels allowing either fluid to flow or gel scaffolds to be injected, mimic the *in vivo* environments allowing *in vitro* observations of both the individual cells and the vascular structure. Unlike the traditional 2D *in vitro* systems where the angiogenic networks do not include vessels/lumens for blood to flow through, the cells have been proved to migrate in a 3D volume in the MFDs to form 3D angiogenic lumens [11, 20]. The microfluidic platform also provides a well-controlled cell culture environment which can closely represent the *in vivo* situations and can be observed easily in real time [44]. As controlled reactions can be reproduced with a small volume of samples and reagents, MFDs can be used nowadays for long-term studies of many biological processes including angiogenesis.

Fig 1(b) illustrates the internal structure of the MFD used in our experiments, where channel A, B, and C are media filling channel, cell seeding channel, and gel-filling channel respectively. Each MFD has 37 sprouting regions separated by posts. These posts are designed to cage the collagen gel (when injected) inside the gel channel. They are also used as features for registering the experimental image sequences. Fig 1(c) illustrates an individual sprouting region.

MFDs are made of Polydimethylsiloxane (PDMS—Sylgard 184 at a ratio of 10:1 polymer to cross-linker, Dow Corning Corporation) in the negative patterned wafers designed by Farahat et al. [11]. The devices are then detached from the wafers, autoclaved in deionized water, and dried overnight in an oven at 70 degrees. Next, the devices are plasma bonded to 22*mm* × 60*mm* sterile glass slides to form closed microfluidic channels (channel A, B, and C in Fig 1(b)). These channels are coated with poly-d-lysine (PDL—P7886, Sigma) for four hours, washed with sterile water, and dried overnight to render the hydrophilic channel surface hydrophobic. The trapezoidal posts in channels allow the filled gel to be confined within the gel channel. Type I collagen (354236, BD Biosciences) is injected into the gel-filling channel and polymerized for one hour in a humidity box in incubator. After filling the media filling channel and cell seeding channel of the MFDs with Endothelial Growth Media (Lonza EGM-2MV Cat. No. CC-3202) [44], these devices are ready for cell seeding.

HMVECs (Lonza Cat. No. CC-2543) are used in our angiogenic experiments. During cell seeding, channel B is filled with the cell suspension which is a mixture of HMVECs and EGM-2MV. These cells will attach to the collagen gel and form a monolayer. Channel A is filled with EGM-2MV only. After HMVECs are confluent in channel B, condition media are filled in channel A and B to initialize angiogenesis. As ECs tend to migrate towards a higher gradient of Vascular Endothelial Growth Factors (VEGF), we create a VEGF gradient across the gel by providing media with higher VEGF concentration in channel A than in channel B. About two days after cell seeding, ECs in the monolayer specialize into tip and stalk cells, migrate out, and form new angiogenic sprouts.

**Fig 2:**
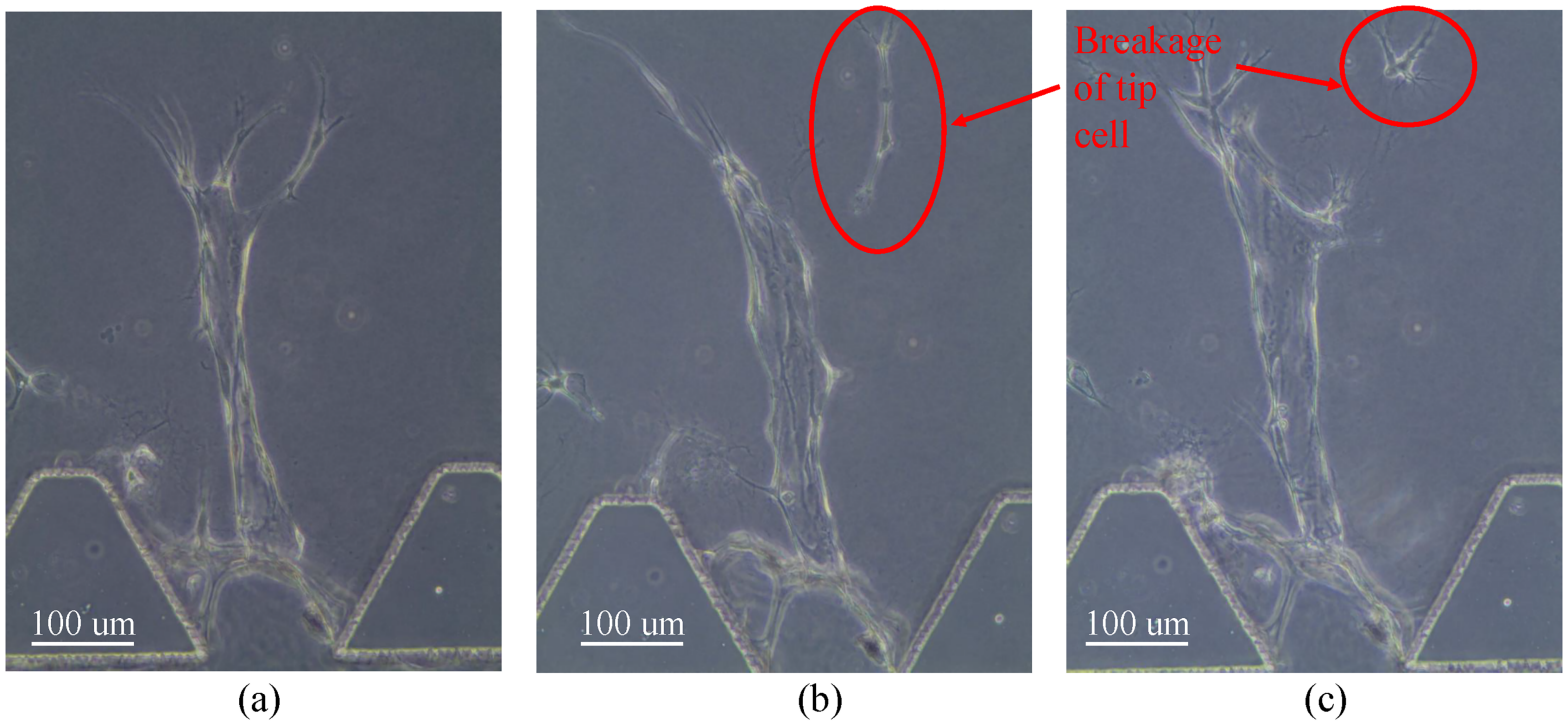
Breakage of tip cell under a high S1P concentration.

S1P has been shown to increase the average vessel length by increasing the migration of HUVECs in [14] and [15]. Therefore, we add S1P in our angiogenic experiments. We started with a S1P concentration of 250nM in channel A, which is same as the concentration used in [15]. Fig 2 provides an example of the angiogenic vessels for 3 consecutive days, from which we can see that adding S1P does increase the vessel length. However, we also observed that when some tip cells migrate too fast, the stalk cells are unable to follow. As a result, the tip cells break away from the sprouts, as labeled in Fig 2. We hypothesize that S1P functions mainly on tip cells and HMVECs are more responsive to S1P than HUVECs. We reduce the S1P concentration to 31nM in order to form long angiogenic vessels with negligible breakage of tip cells.

By varying the S1P concentrations in the condition media filled in channel A and channel B, we create three different S1P experimental conditions, as shown in Table 1. We consider these three S1P conditions to further study the influence of S1P on angiogenic vessel morphologies. For all experiments, we apply a fixed VEGF condition (40 ng/ml in channel A and 20 ng/ml in channel B).

**Table 1:** Varied S1P conditions in the angiogenic experiments.

Experiment conditions	S1P conc. in channel A (ng/ml)	S1P conc. in channel B (ng/ml)
**No S1P (ctrl)**	0	0
**Positive S1P gradient**	31	0
**Negative S1P gradient**	0	31

To avoid the inhibition of cell proliferation and migration due to staining [45], we culture the unstained ECs and observe the vessels with a phase contrast microscope [46] (at 20x magnification) daily over a period of ten to fourteen days, as illustrated in Fig 1(d). At the end point, we image the sprouts by a confocal microscope (see Fig 1(e)) after staining cell nuclei with Hoechst 33342 (Invitrogen Cat. No. H1399) and actin with Phalloidin 488 (Invitrogen Cat. No. A12379).

## Automated vessel formation tracking system

**Fig 3:**
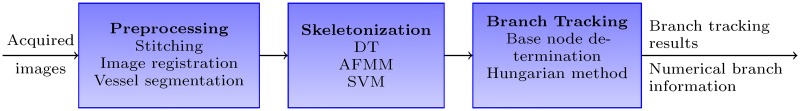
Diagram of the automated vessel formation tracking system. Preprocessing to extract binary vessel shapes. Skeletonization to obtain the connected skeleton segments with branch nodes and end nodes. Branch tracking to identify the routes for the main branches and associate the branches over time.

**Fig 4:**
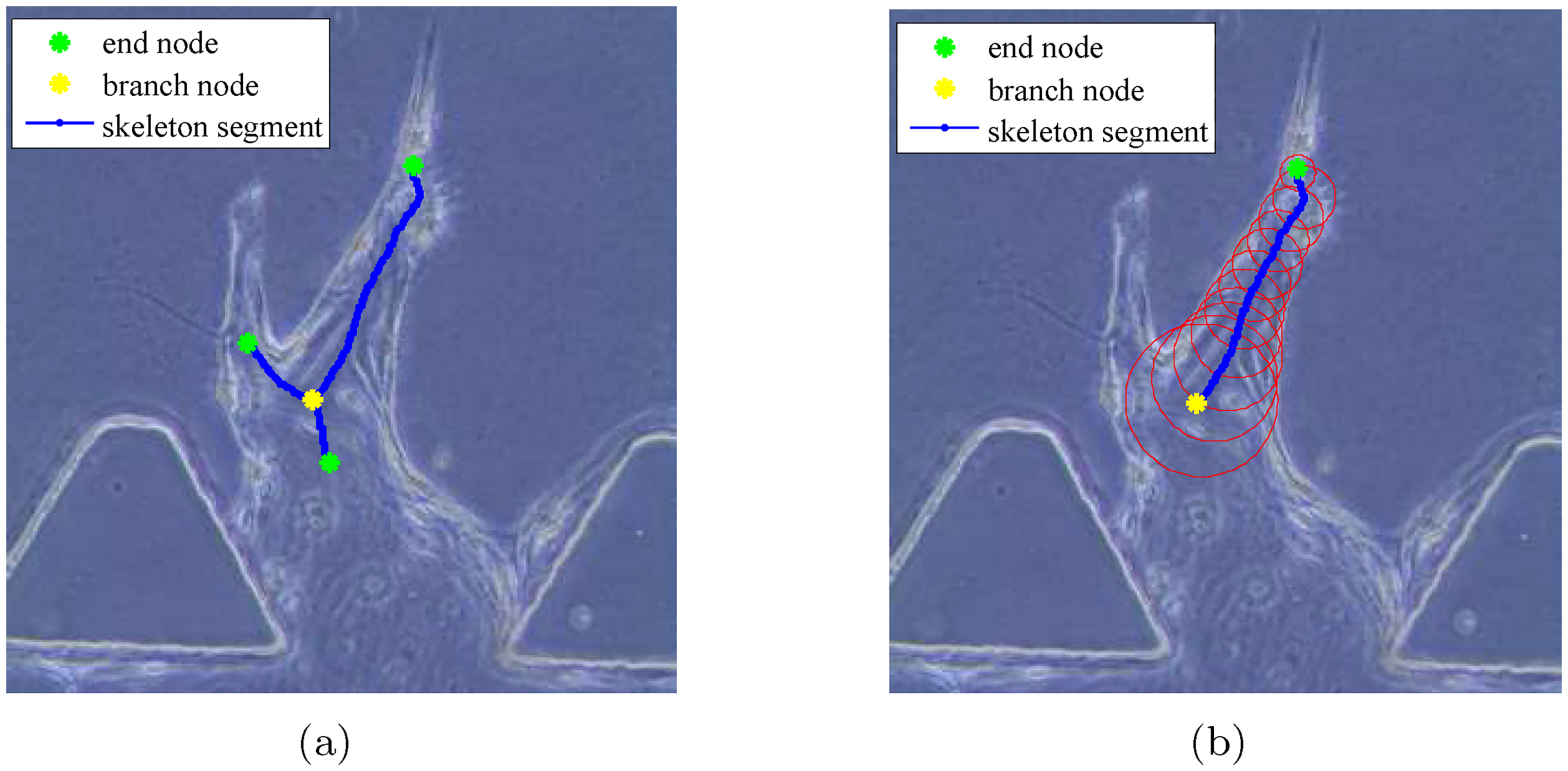
Example of skeleton segments and nodes. (a) Skeleton segments and nodes. (b) One segment. Branch nodes in yellow, end nodes in green, segments in blue, and vessel radii in red.

Fig 3 illustrates the proposed automated vessel formation tracking system (AVFTS), which consists of preprocessing, skeletonization, and branch tracking. The first step is to extract binary vessel shapes by preprocessing the experimental phase contrast image sequences through stitching, image registration, and vessel segmentation. Skeletonization, providing a simple representation of the vessel shape, is a crucial step in this system. We apply the distance transform (DT) and an augmented fast marching method (AFMM) on the binary vessel shapes to extract the skeletons of the angiogenic vessels. Fig 4(a) shows one example of the extracted skeletons composing of a set of nodes and segments. Specifically, the yellow star is a branch node and the 3 green stars are end nodes. The blue lines connecting two nodes are defined as segments. Each branch node connects to at least 3 other neighboring nodes by segments, and each end node connects to only 1 neighboring node. Fig 4(b) illustrates an individual segment with branch node (yellow star), end node (green star), segment (blue line), and the vessel radii (red circles). Next, we label each segment by a trained SVM classifier in order to remove the specious segments which are not part of vessels. Lastly, we determine the base nodes, identify the routes for the main branches by joining the connected segments, and apply the Hungarian method to associate the branches over time in order to obtain the branch trajectories. The geometric parameters for the angiogenic vessels including the average vessel length/width, the number of branches, and the number of nodes are also extracted. The AVFTS is implemented via custom scripts in MATLAB (Mathworks Inc.).

In the following, we will explain the three modules of the AVFTS: preprocessing, skeletonization, and branch tracking.

### Preprocessing

There are three major steps in the preprocessing, leading to the binary vessel shapes: stitching, image registration, and vessel segmentation.

#### Stitching

**Fig 5:**
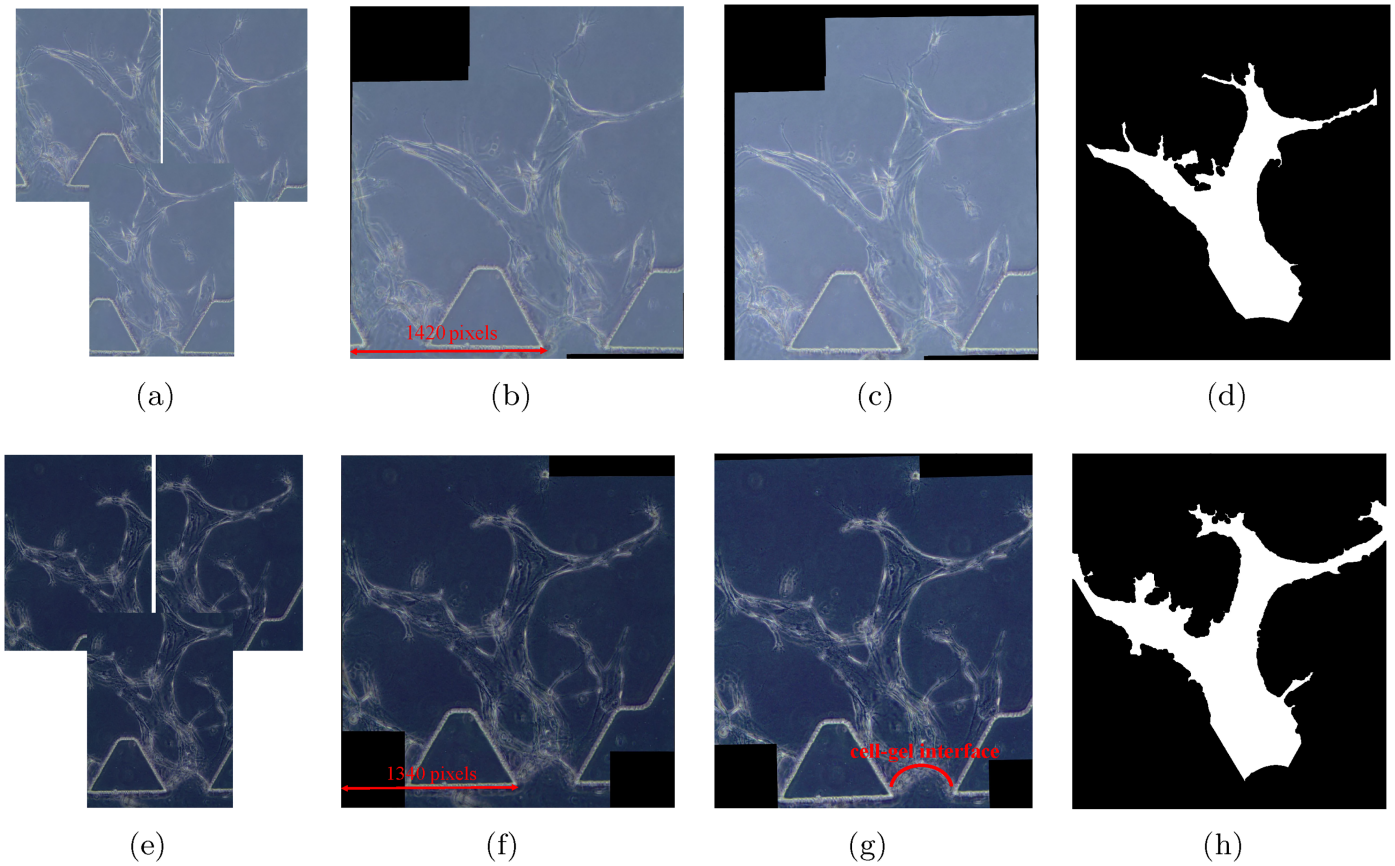
Preprocessing results for angiogenic vessels in a same sprouting region over 2 consecutive days. (a) Acquired images (day 11). (b) Stitched image (day 11). (c) Aligned image (day 11). (d) Binary vessel shape (day 11). (e) Acquired images (day 12). (f) Stitched image (day 12). (g) Aligned image (day 12). (h) Binary vessel shape (day 12).

Due to imaging constraints, several overlapping images are required to capture a long vessel in a sprouting region at 20x magnification. In Fig 5(a) and 5(e), we provide examples of overlapping phase contrast images of angiogenic vessels in the same sprouting region over 2 consecutive days (day 11 and day 12 respectively).

Our first step is to construct a full view of the long angiogenic vessels by stitching the overlapping images. Speeded Up Robust Features (SURF) are employed as the features to find and match the corresponding points for stitching [47]. The stitched results of the overlapping images in Fig 5(a) and 5(e) are shown in Fig 5(b) and 5(f) respectively.

#### Image registration

We acquired images manually at different time. Therefore, the angiogenic vessels are not aligned across the image sequences. The images in Fig 5(b) and 5(f) show that there is a difference in the locations of the posts. We use these posts as features to spatially align the time-lapse images of each sprouting region. We detect and represent the post edges by the Hough Transform (HT) [26]. With the posts as the correspondences among the image sequences, we derive the scale, linear translation, and rotation parameters to align all the image sequences to one coordinate system. Fig 5(c) and 5(g) show the registered images for the examples in Fig 5(a) and 5(e) respectively. This step is critical for tracking the vessel formation.

#### Vessel segmentation

Since the angiogenic vessels form in the collagen gel, we are mostly interested in the gel region, which is bounded by the posts and the cell-gel interface (see Fig 5(g)). In order to remove the non-gel region, we first determine the boundaries of the gel region by detecting the posts and the cell-gel interface. The posts are detected and represented by the HT in image registration step. The cell-gel interface in the experimental images can be estimated by fitting a parabola. After masking out the pixels in the identified posts region and below the cell-gel interface, we convert the experimental images into binary images with the global image threshold computed using Otsu’s method [48]. We obtain the segmented vessel shapes through a series of morphological image processing steps, including closing to fill the holes inside the vessel area, erosion to reduce noise size, and area opening to remove the noise [49]. The segmented binary vessel shapes for the examples in Fig 5(a) and 5(e) are shown in Fig 5(d) and 5(h) respectively.

### Skeletonization

After obtaining the binary vessel shapes through preprocessing, skeletonization is applied to quantitatively represent the vessel shapes.

Firstly, we apply a skeletonization technique based on DT and AFMM to extract the connected skeleton segments with branch nodes and end nodes (see Fig 4(a)). We then train an SVM classifier in order to label and remove the specious segments which are due to noise or part of filopodia.

#### Distance transform and augmented fast marching method for skeletonization

Our skeletonization approach relies on an accurate distance map that can be generated by the DT [36]. For a given object *Ω* with the boundary *δΩ*, DT labels each point *p* of the object by the distance to its closest point *q* on the boundary. For all the points *p* ∈ *Ω*, DT can be defined by [50]:

$$\begin{array}{c} T\left(p\right)=\min_{q\in \delta \Omega }\left(\text{dist}\left(p,q\right)\right), \end{array}$$ (1)

where dist(*p*, *q*) is the distance metric. Specifically, Euclidean distance is employed as the distance metric in this paper. It is computed by:

$$\begin{array}{c} \text{dist}\left(p,q\right)={∥p-q∥}_{2}. \end{array}$$ (2)

DT in Eq (1) can be computed with FMM by evolving the boundaries in normal direction [41]:

$$\begin{array}{c} |\nabla T|=1. \end{array}$$ (3)

**Fig 6:**
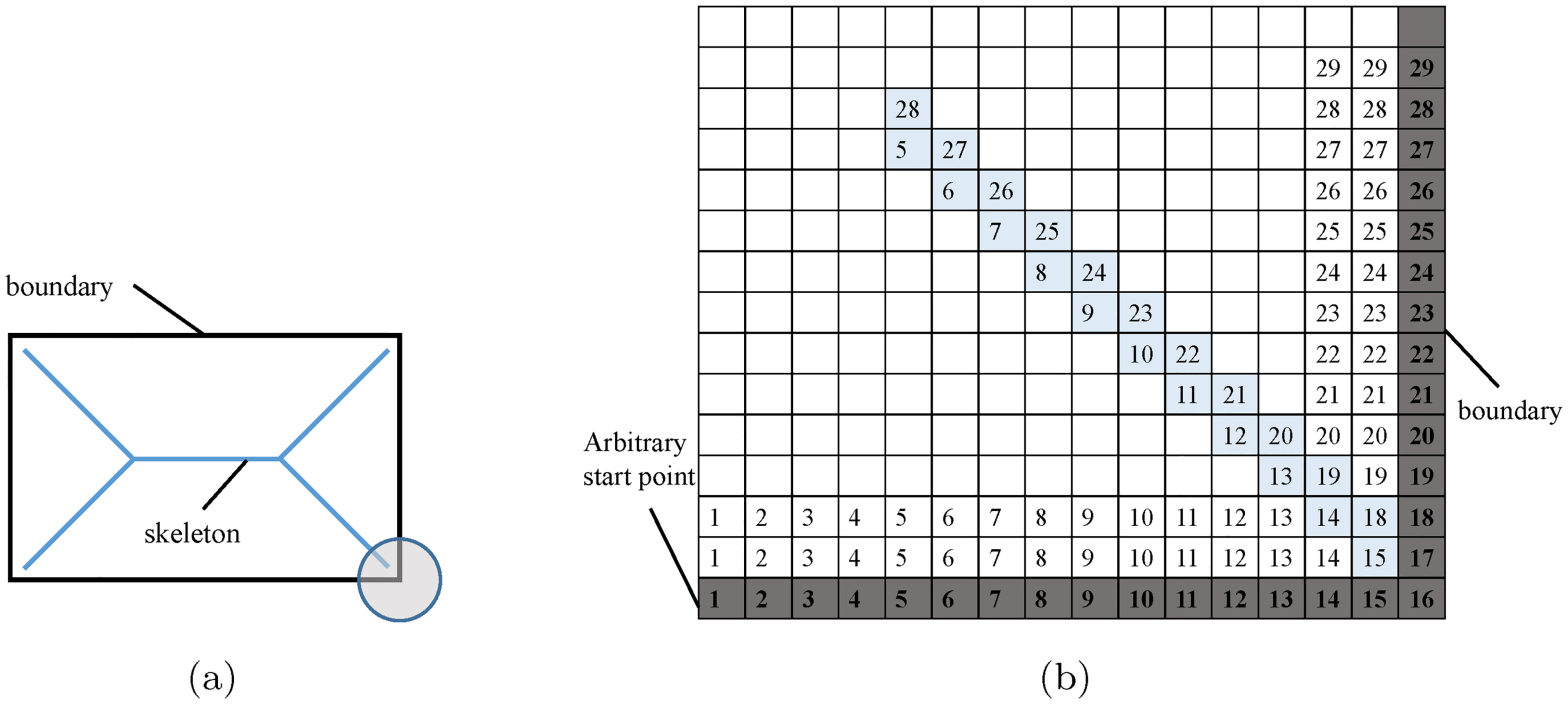
Demonstration of AFMM algorithm to find the skeleton of a simple rectangular shape. (a) Skeleton of a rectangular. (b) *U* values of the lower right corner of the rectangular.

The skeleton lies along the singularities in the DT. To initialize the FMM, *T* is set to 0 for all the points on the boundary *δΩ* and inf for all the points inside the object *Ω*. Eq (3) can be solved by finite difference discretization on a Cartesian grid [42]. However, direct computation of the singularities in the DT is difficult. To compute the skeleton, the FMM algorithm is augmented by one real value *U* for each pixel point [41]. For the pixel on the boundary *δΩ*, we choose an arbitrary pixel to set its *U* = 1 and then monotonically increase *U* along the boundary. For each pixel inside the object *Ω*, the value *U* indicates the parameterized boundary location that it came from, which is obtained from the DT. Fig 6(a) shows the skeleton of a rectangular and Fig 6(b) illustrates how the *U* values are computed by AFMM for the lower right corner of the rectangular.

After computing the *U* values for all the pixel points, the skeleton can be obtained by detecting the sharp discontinuities in *U*. In other words, if the *U* value for one point differs from the *U* values of its neighboring points by more than a given threshold, it will be retained as a point in the skeleton. In Fig 6(b), the pixels on the diagonal line labeled in light blue are extracted as skeleton.

**Fig 7:**
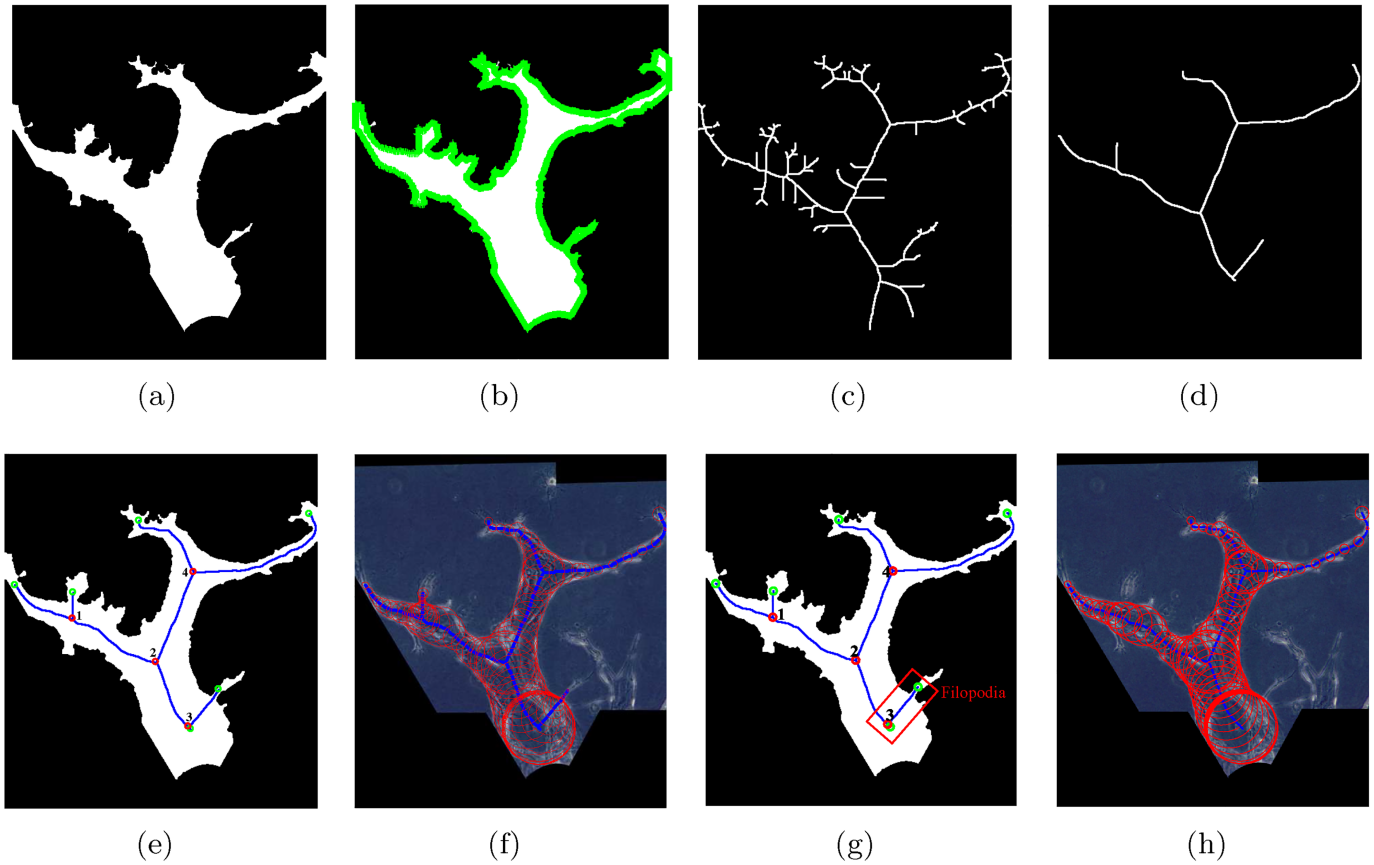
An example of skeletonization result. (a) Binary vessel shape. (b) Boundary of the binary vessel. (c) Skeletons from thinning. (d) Skeletons from DT and AFMM. (e) Skeleton segments with nodes. (f) Skeleton segments with radii in the original phase contrast image. (g) An example of a filopodia wrongly labeled as skeleton. (h) Skeleton segments with radii after SVM classification.

Fig 7(a) shows an example of the binary vessel shape obtained from the preprocessing step. Computation of the vessel skeleton requires the boundary coordinates of the vessel itself. Thus, we first acquire the vessel boundary by applying the “bwboundaries” function in MATLAB, based on the Moore-Neighbor tracing algorithm [51]. In Fig 7(b), we plot the extracted vessel boundary in green.

For “noisy” vessel boundaries, applying a standard skeletonization based on iterative thinning algorithm [52] results in a large number of noisy protrusions, as shown in Fig 7(c). We apply the AFMM algorithm to compute the skeleton of the binary vessel shape [41]. By selecting a proper threshold of the *U* value to remove noisy protrusions, we extract the connected skeleton segments, as shown in Fig 7(d). By comparing Fig 7(d) with 7(c), it is clear that the vessel skeleton obtained with the AFMM algorithm is less sensitive to noise in the boundary.

Fig 7(e) shows the connected skeleton segments and nodes in the binary vessel shape, where the 4 branch nodes are plotted as red circles and the 6 end nodes are plotted as green circles. In order to compute the vessel width, we divide each segment into *N*_*eq*_ − 1 equidistant parts and estimate the radii at these *N*_*eq*_ points by their minimum distances to the boundaries. A larger *N*_*eq*_ leads to more accurate estimation of the vessel width but at the expense of computation time. Therefore we choose *N*_*eq*_ = 11 empirically. In Fig 7(f), we plot the skeleton segments with radii in the original phase contrast image.

#### Support vector machines for segment classification

Although the skeletonization by DT and AFMM is less sensitive to boundary noise compared with morphological thinning, there are still some specious skeleton segments that are caused by noise or one part of filopodia. By comparing the skeleton segments in the phase contrast images with their 3D confocal images of the stained vessels, we confirm whether a segment represents the angiogenic vessels or otherwise. For example, in Fig 7(g), we can identify that the skeleton segment in the red rectangle is one part of the filopodia. In other words, the red node 3 in Fig 7(g) is not a true branch node since it only connects with two skeleton segments that belong to a vessel. However, further increase of the threshold would lead to the loss of the main skeleton segments.

The number of branch nodes is important for estimating the number of branches. The critical feature for a branch node is that it has at least 3 connected skeleton segments that belong to a vessel. In order to decide whether a node is a branch node, we train a binary classifier, specifically, a binary SVM classifier, to remove the specious skeleton segments.

SVMs are a set of supervised learning methods used for classification, regression, and outlier detection [53]. Soft-margin SVM is applicable to classify linearly inseparable data by an optimal hyperplane, which allows for a tolerable misclassification rate. The decision function for a soft-margin SVM is given by [54]:

$$\begin{array}{c} D\left(x^{i}\right)=w^{T}x^{i}+b, \end{array}$$ (4)

where **x**^*i*^ is the *i*th template, *w* is the normal vector of the hyperplane, and *b* is a bias term.

In order to obtain the values of *w* and *b* for the optimal hyperplane, we first prepare data to train the binary SVM classifier. We preprocess the experimental phase contrast images to get the binary vessel shapes, and then apply DT and AFMM to extract the skeleton segments with radii.

**Fig 8:**
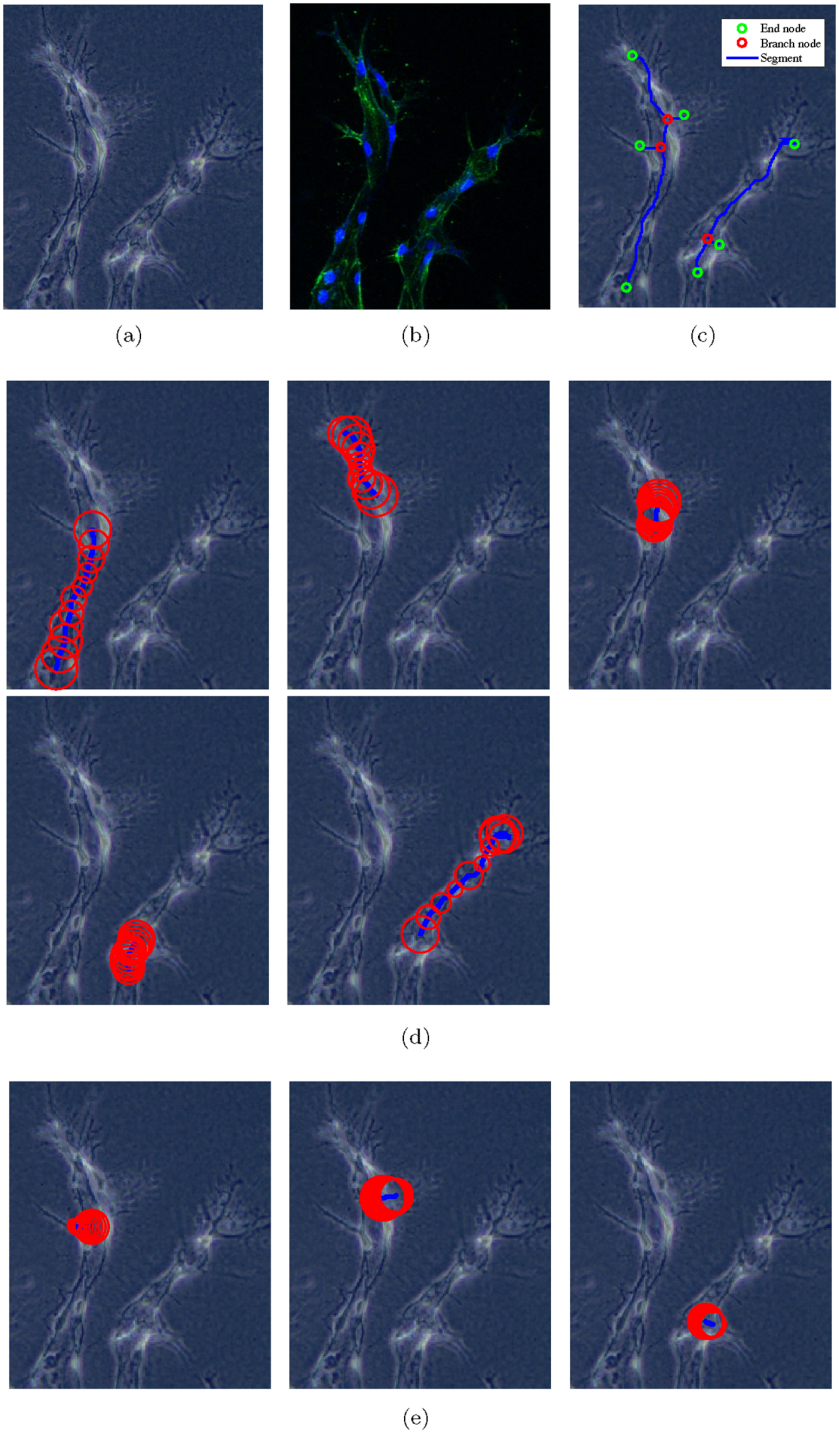
Examples of training templates for the binary SVM classifier. (a) and (b) are vessels in the phase contrast image and confocal image respectively. (c) shows skeleton segments and nodes from DT and AFMM. (d) are positive templates and (e) are negative templates.

Fig 8 shows an example of how we obtain the training templates. The image in Fig 8(a) is cropped from the experimental phase contrast image, and Fig 8(b) is its corresponding confocal image. Fig 8(c) shows the skeleton segments and nodes obtained from DT and AFMM. By comparing with Fig 8(b), we label each skeleton segment in Fig 8(c) as a positive or negative training template. The positive templates and negative templates are displayed in Fig 8(d) and 8(e) respectively. For each skeleton segment *i*, its label *y*^*i*^ is defined as:

$$\begin{array}{cc} y^{i} & =\{\begin{array}{cc} 1 & \;\;\;\;\text{if}\;\text{skeleton}\;\text{segment}\;\text{belongs}\;\text{to}\;\text{a}\;\text{vessel}\\ -1 & \;\;\;\;\text{otherwise} \end{array}. \end{array}$$ (5)

The features we extract for each training template include:

- Segment length *l*: a scalar value which measures the length of the skeleton segment,
- Segment radii **r**: a vector containing a set of radii acquired at the equidistant points in the skeleton segment,
- Maximum radius *r*_*max*_: maximum value of the segment radii **r**,
- Minimum radius *r*_*min*_: minimum value of the segment radii **r**,
- Connection type *J*: a scalar value based on the two nodes connected by the skeleton segment which includes: branch-branch node connection, branch-end node connection, and end-end node connection.

We aim to find the best combination of the above features to train the binary SVM classifier. Our training data includes 214 positive training samples and 102 negative training samples. After testing every possible combination of features, we obtain the most appropriate feature vector as follows:

$$\begin{array}{cc} x^{i} & =[\begin{array}{c} \;l^{i}\;\\ \;r_{max}^{i}\;\\ \;r_{min}^{i}\;\\ \;J^{i}\; \end{array}], \end{array}$$ (6)

for the *i*th training template. We obtain 84% accuracy in 5-fold cross-validation when training the SVM classifier with the feature vector in Eq (6).

We apply the trained SVM classifier to label and remove the non-vessel segments from the image. Fig 7(h) shows the final skeleton with vessel radii after SVM classification.

For each node, the number of the segments it connects to is updated accordingly. The nodes whose updated number of connected segments is equal to or larger than three are labeled as the final branch nodes. The nodes with two connected segments are called junction nodes.

### Branch tracking

#### Base nodes determination

We obtain all the skeleton segments with radii in skeletonization step. However, it is not clear which segments belong to which individual vessel or branch of a vessel. Consequently, to track the vessel formation, we first need to join the linked skeleton segments and identify the route (the whole centerline) of each branch.

**Fig 9:**
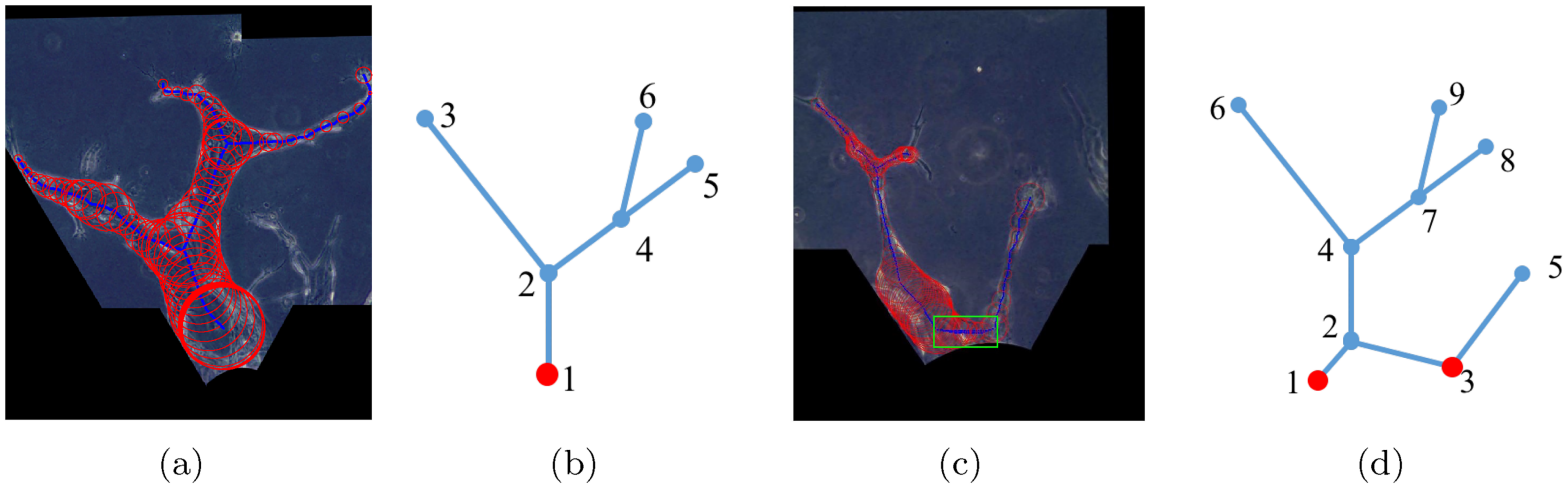
Base nodes determination for the two different scenarios. (a) and (c) are the skeletons with radii in the experimental images for the two scenarios respectively. (b) and (d) are their corresponding generalized schematic diagrams where the base nodes are plotted in red.

There are generally two scenarios for the skeletons, as illustrated in Fig 9(a) and 9(c). Their generalized schematic diagrams are depicted in Fig 9(b) and 9(d) respectively. We define the base node as the node where a branch starts with. Generally, the branches that originate from one same tip cell will share a same base node.

In Scenario 1, all the branches are originally from a same tip cell. This scenario is quite straightforward. The node which is nearest to the monolayer is selected as the base node, which is plotted in red in Fig 9(b).

In Scenario 2, the vessels are originally from two different tip cells. However, they are connected by a artificial segment (located in the green box in Fig 9(c)) that is part of the cell-gel interface segmented. In order to determine the base nodes, we need to detect and remove the skeleton segment which connects the two parts. The two base nodes are plotted in red in Fig 9(d). Algorithm 1 demonstrates how we determine the scenario so as to find the base nodes.

**Algorithm 1** Base nodes determination

 Find the node A nearest to the monolayer

 **if** A is an end node **then**

  Check whether A has nearest branch node

  **if** No **then**

   A is the base node (Scenario 1)

  **else**

   Find the branch node B and its other two neighboring nodes C and D

   **if** ratio_CB_ > *τ* AND ratio_DB_ > *τ* **then**

    A is the base node (Scenario 1)

   **else if** ratio_CB_ > *τ* AND ratio_DB_ < *τ* **then**

    A and D are the base nodes (skeleton segments between node B and D will be removed)

   **else if** ratio_CB_ < *τ* AND ratio_DB_ > *τ* **then**

    A and C are the base nodes (skeleton segments between node B and C will be removed)

   **end if**

  **end if**

 **else if** A is a junction node **then**

  Find its two neighboring nodes E and F

  **if** ratio_EA_ > *τ* AND ratio_FA_ > *τ* **then**

   A is the base node

  **else if** ratio_EA_ > *τ* AND ratio_FA_ < *τ* **then**

   A and F are the base nodes (skeleton segments between node A and F will be removed)

  **else if** ratio_EA_ < *τ* AND ratio_FA_ > *τ* **then**

   A and E are the base nodes (skeleton segments between node A and E will be removed)

  **end if**

 **end if**

Based on the observations of the experimental images, the angle between the monolayer and the artificial skeleton segment which connects two parts is small. To account for this angle, for a skeleton segment with node M and N, the parameter ratio is estimated:

$$\begin{array}{c} {\text{ratio}}_{\text{MN}}=\frac{|y_{N}-y_{M}|}{|x_{N}-x_{M}|}, \end{array}$$ (7)

where (*x*_*M*_, *y*_*M*_) and (*x*_*N*_, *y*_*N*_) are the coordinates of node M and N respectively. The ratios in Algorithm 1 can be estimated by Eq (7). By comparing the ratios with a predefined threshold *τ* and following Algorithm 1, the base nodes and the final skeleton segments are determined.

With the diagram in Fig 9(d) as an example, after base nodes determination, we have:

- Base nodes: node 1 and 3,
- Branch nodes: node 4 and 7,
- End nodes: node 5, 6, 8, and 9,
- Segments: [1 2], [2 4], [4 6], [4 7], [7 8], [7 9], and [3 5],

where each segment is represented by the two nodes it connects to.

Next, we connect up each segment to complete a single branch using the MATLAB function “graphshortestpath”. Each branch starts with a base node and ends with an end node. Matlab function “graphshortestpath” finds the shortest path from one node to the other node in the graph [55]. By providing the base nodes, the end nodes, and the segments, we identify the routes for all the branches. The route for each branch is indicated by the nodes it passes by. For example, there are four branches in Fig 9(d) and each of them is represented by:

- Branch 1: [1 2 4 6],
- Branch 2: [1 2 4 7 8],
- Branch 3: [1 2 4 7 9],
- Branch 4: [3 5].

**Fig 10:**
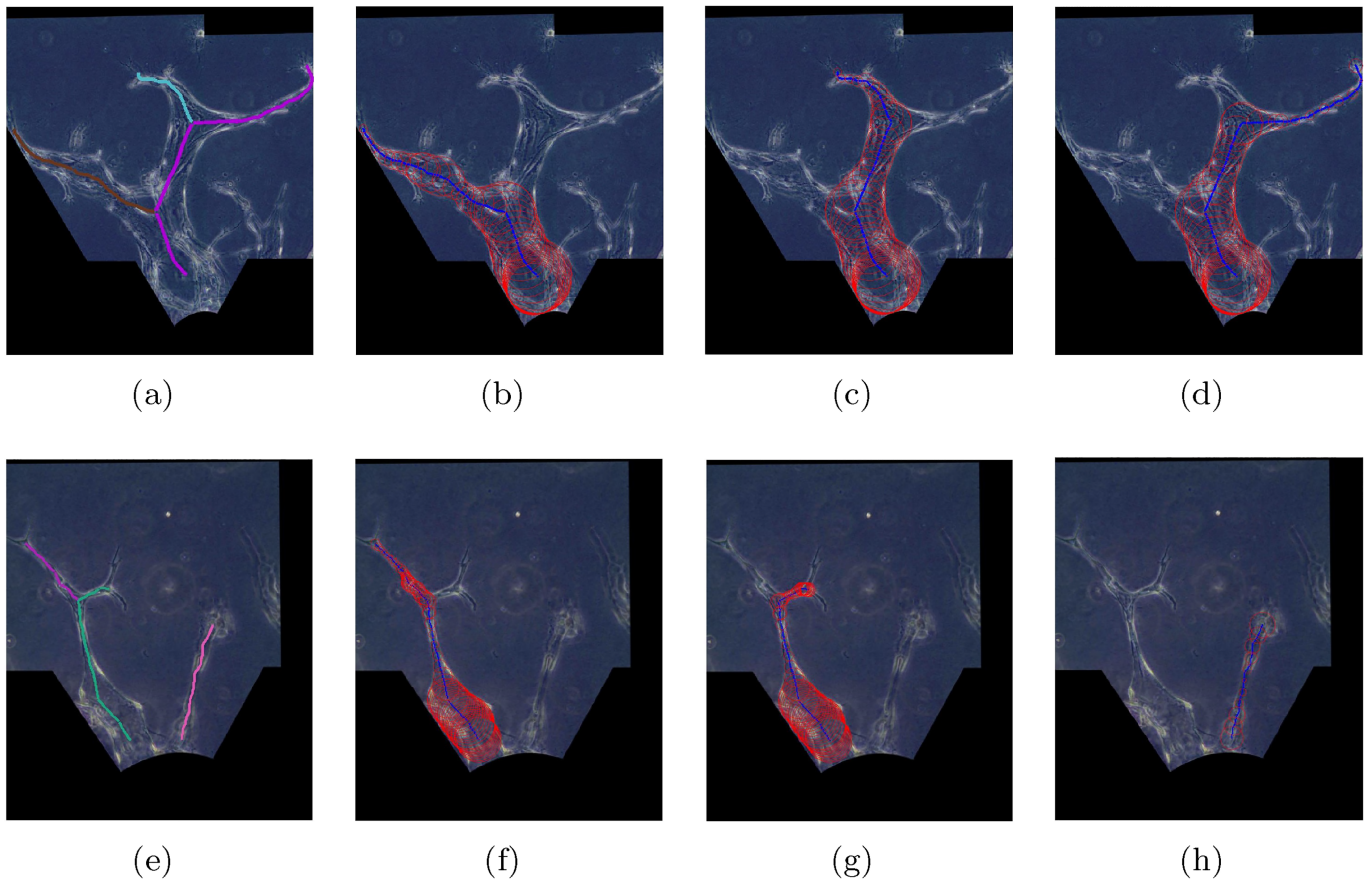
Examples of identified branch routes. (a) and (e) show the identified routes for the examples in Fig 9(a) and 9(c) respectively. Each branch of the vessels is plotted in (b) to (d) and (f) to (g), where the blue lines are the continuous skeletons and the red circles are the branch radii at selected points in the skeletons.

Fig 10(a) and 10(e) show the identified branch routes for the examples in Fig 9(a) and 9(c) respectively, where each color represents one branch. Specifically, each branch of the vessels in Fig 10(a) is plotted in Fig 10(b) to 10(d), and each branch of the vessels in Fig 10(e) is plotted in Fig 10(f) to 10(h), where the blue lines are the continuous skeletons and the red circles are the branch radii at selected points in the skeletons.

As illustrated in Fig 10, the quantitative values of the vessel length/width and number of branches in each sprouting region are also extracted from the experimental images.

#### Branch association over time

**Fig 11:**
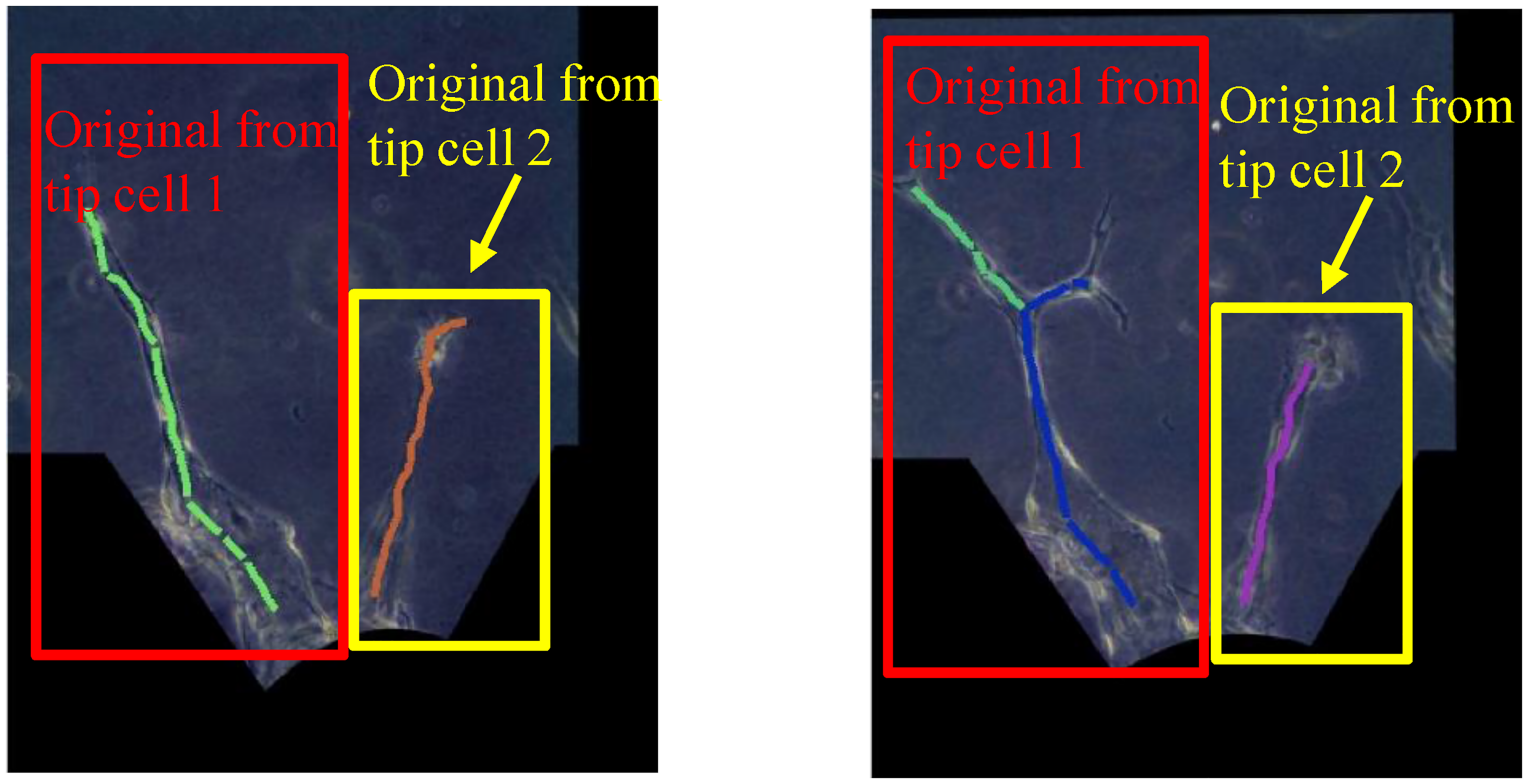
Skeletons of the angiogenic vessels over two consecutive days. The sprouts in the red rectangular box and in the yellow box are originally from two different tip cells that migrated from the monolayer.

After identifying the route for each branch, the next step is to track the branch formation over time. All the branches from a same tip cell that migrated from the monolayer share a same base node. All the branches sharing a same base node are regarded as a sprout. Fig 11 shows the skeletons of the angiogenic vessels over two consecutive days. Each image in Fig 11 contains two angiogenic sprouts, which are within the red rectangular box and yellow rectangular box respectively. Over time sequence, the sprout within the red box are from same tip cell (i.e. cell 1) and the sprout within the yellow box are from same tip cell (i.e. cell 2). We first associate the sprout as a whole and then associate branches in each sprout respectively over time.

#### Step 1: Associate angiogenic sprouts over time

Each sprout in an image is regarded as a whole when matching the sprouts over time. From the experimental observations, two different sprouts generally do not overlap each other. In other words, for different sprouts, their skeletons differ significantly in the average *x*-positions. Therefore, we associate the sprouts by comparing their average *x*-positions with the following steps:

- Compute the number of sprouts in frame *t* + 1,
- Check whether there are new sprouts by comparing with the number of the sprouts in the frame *t*,
- Match the existing sprouts and determine the new sprouts based on their average *x*-positions by the “nearest-neighborhood” algorithm.

#### Step 2: Associate branches in each sprout over time

After associating the sprouts, we match the branches in each associated sprout over time. We start with tracking the branch nodes based on their relative locations. Suppose we have *n*_1_ branch nodes in one sprout at frame *t* and *n*_2_ branch nodes in its corresponding sprout at frame *t* + 1. The pairwise Euclidean distance matrix for the branch nodes in both sprouts $C\in R^{n_{1}\times n_{2}}$ is defined as:

$$\begin{array}{c} C=[\begin{array}{ccc} \;c_{1,1}\; & \;\cdots \; & \;c_{1,n_{2}}\;\\ \;\vdots \; & \;\ddots \; & \;\vdots \;\\ \;c_{n_{1},1}\; & \;\cdots \; & \;c_{n_{1},n_{2}}\; \end{array}]. \end{array}$$ (8)

**C** is a cost matrix to distinguish the new branch nodes and match the existing branch nodes.

The Hungarian method, a combinatorial optimization algorithm proposed by [56], is applied to associate the branch nodes. However, as explained in Theorem 1 and Theorem 2, it is only applicable to a square matrix. For our case, the number of branch nodes changes over time when new branches form or existing branches retract. In other words, **C** is not a square matrix when *n*_2_ ≠ *n*_1_. Since retractions are rarely observed in between the imaging time-frame in our experiments, we assume that the number of branch nodes changes only when new branches form. Specifically, only *n*_2_ ≥ *n*_1_ is considered.

We create a new square matrix $P\in R^{n_{2}\times n_{2}}$. If *n*_2_ > *n*_1_, **P** is obtained by adding *n*_2_ − *n*_1_ rows to **C**:

$$\begin{array}{c} p_{i,j}=\{\begin{array}{cc} c_{i,j} & \;\;\;\;\;\;\;\;\;\;\text{for}\;\;i=1:n_{1}\;\;\text{and}\;\;j=1:n_{2}\\ c_{0} & \;\;\;\;\text{for}\;\;i=n_{1}+1:n_{2}\;\;\text{and}\;\;j=1:n_{2} \end{array}, \end{array}$$ (9)

where *c*_0_ is an assigned constant value that is larger than all the other distances. If *n*_2_ = *n*_1_,

$$\begin{array}{c} p_{i,j}=c_{i,j}\;\;\;\;\text{for}\;\;i=1:n_{2}\;\;\text{and}\;\;j=1:n_{2}. \end{array}$$ (10)

We introduce an *n*_2_ × *n*_2_ matrix **Q** to optimize the matching of the branch nodes by minimizing:

$$\begin{array}{c} \sum_{i=1}^{n_{2}}\sum_{j=1}^{n_{2}}p_{i,j}q_{i,j}, \end{array}$$ (11)

subject to the constraints:

$$\begin{array}{c} \sum_{i=1}^{n_{2}}q_{i,j}=1,\;\;\text{for}\;\;j=1,\cdots ,n_{2}, \end{array}$$ (12)

$$\begin{array}{c} \sum_{j=1}^{n_{2}}q_{i,j}=1,\;\;\text{for}\;\;i=1,\cdots ,n_{2}. \end{array}$$ (13)

The conditions in Eqs (12) and (13) enforce that all elements in **Q** are zeros except for one 1 in each row and column, guaranteeing a one-to-one matching.

The Hungarian method is rooted in the following two Theorems. Through Theorem 1, the matrix **P** is transformed into another matrix $\hat{P}$, which contains an independent set of *n*_2_ zeros. Theorem 2 generates the optimal solution **Q** for the association [56–58].

**Theorem 1** *A solution* **Q** *minimizes*

$$\begin{array}{c} \sum_{i=1}^{n_{2}}\sum_{j=1}^{n_{2}}p_{i,j}q_{i,j} \end{array}$$ (14)

*if and only if it minimizes*

$$\begin{array}{c} \sum_{i=1}^{n_{2}}\sum_{j=1}^{n_{2}}{\hat{p}}_{i,j}q_{i,j}, \end{array}$$ (15)

*where* ${\hat{p}}_{i,j}=p_{i,j}-u_{i}-v_{j}$, *u*_*i*_ *and v*_*i*_ *are real numbers for all i* = 1, ⋯, *n*_2_ *and j* = 1, ⋯, *n*_2_.

**Theorem 2** *If all the elements in* **P** *satisfy p*_*i*, *j*_ ≥ 0 *and* {*p*_1,*j*_1__, *p*_2,*j*_2__, ⋯, *p*_*n*_2_, *j*_*n*_2___} *is an independent set of n*_2_ *zeros in* **P**, *then the matrix* **Q** *with*

$$\begin{array}{c} q_{1,j_{1}}=1, \end{array}$$ (16)

$$\begin{array}{c} q_{2,j_{2}}=1,\\ \vdots \end{array}$$ (17)

$$\begin{array}{c} q_{n_{2},j_{n_{2}}}=1, \end{array}$$ (18)

$$\begin{array}{c} q_{i,j}=0\;\;for\;all\;other\;\;i\;\;and\;\;j, \end{array}$$ (19)

*is the optimal solution to minimize* $\sum_{i=1}^{n_{2}}\sum_{j=1}^{n_{2}}p_{i,j}q_{i,j}$.

We determine the new branch nodes and associate the existing branch nodes from the optimal solution **Q**. For two consecutive frames *t* and *t* + 1, the elements *q*_*i*, *j*_ = 1 in **Q** indicates that at frame *t* + 1:

$$\{\begin{array}{ll} j\;\text{is}\;\text{a}\;\text{new}\;\text{branch}\;\text{node}, & \text{if}\;p_{i,j}=c_{0};\\ j\;\text{corresponds}\;\text{to}\;i\;\text{at}\;\text{frame}\;t, & \text{otherwise.} \end{array}$$

For the case that branch node *j* at frame *t* + 1 corresponds to branch node *i* at frame t, we match the segments connect to *j* and *i* based on their average *x*-positions. For the case that *j* is a new branch node, we label the skeleton segments connected to *j* as new branches.

**Fig 12:**
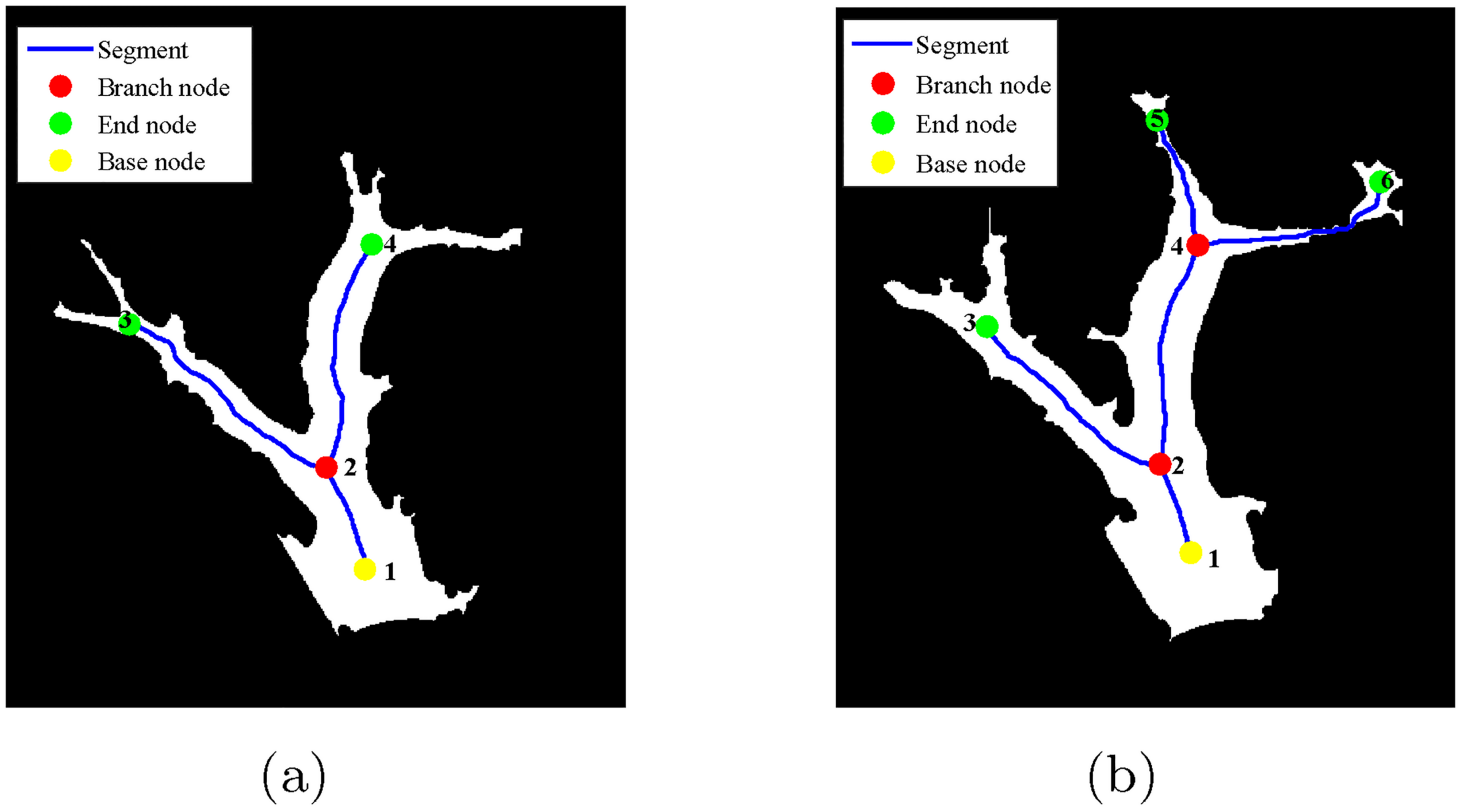
Skeletons and nodes for angiogenic vessels of two consecutive days. (a) Day 9. (b) Day 10.

We use the examples in Fig 12 to demonstrate the segment association. Table 2 lists the route for each branch in Fig 12.

**Table 2:** Branches for the vessels in Fig 12.

	Branches
Day 9	[1 2 3], [1 2 4]
Day 10	[1 2 3], [1 2 4 5], [1 2 4 6]

As shown in Fig 12, branch node 2 in day 9 corresponds to branch node 2 in day 10. The segments connected to the branch node 2 in day 9 are [2 3] and [2 4]. The segments connected to the branch node 2 in day 10 are [2 3] and [2 4]. By comparing the average *x*-positions of these segments, we obtain that segment [2 3] in day 9 corresponds to [2 3] in day 10, and segment [2 4] in day 9 corresponds to [2 4] in day 10. For the new branch node 4 in day 10, the segments [4 5] and [4 6] connected to it are labeled as new branches.

## Results and discussion

### Tracking of vessel formation

By applying the proposed AVFTS, we are able to extract the length and width of the angiogenic vessels based on the coordinates and the radii of the skeleton segments. Moreover, we generate a table to demonstrate the historical information for each branch, from the first day till last day of the experiment, as illustrated in Table 3 (specifically for the example in Fig 12).

**Table 3:** Examples of tracking branch formation. The tracking results assign each numbered skeletal segment to each branch at each day.

Day 1	⋯	Day 9	Day 10	Branch
No vessels	⋯	[1 2 3]	[1 2 3]	**1**
	⋯	[1 2 4]	[1 2 4 5]	**2**
			[1 2 4 6]	**3**

**Fig 13:**
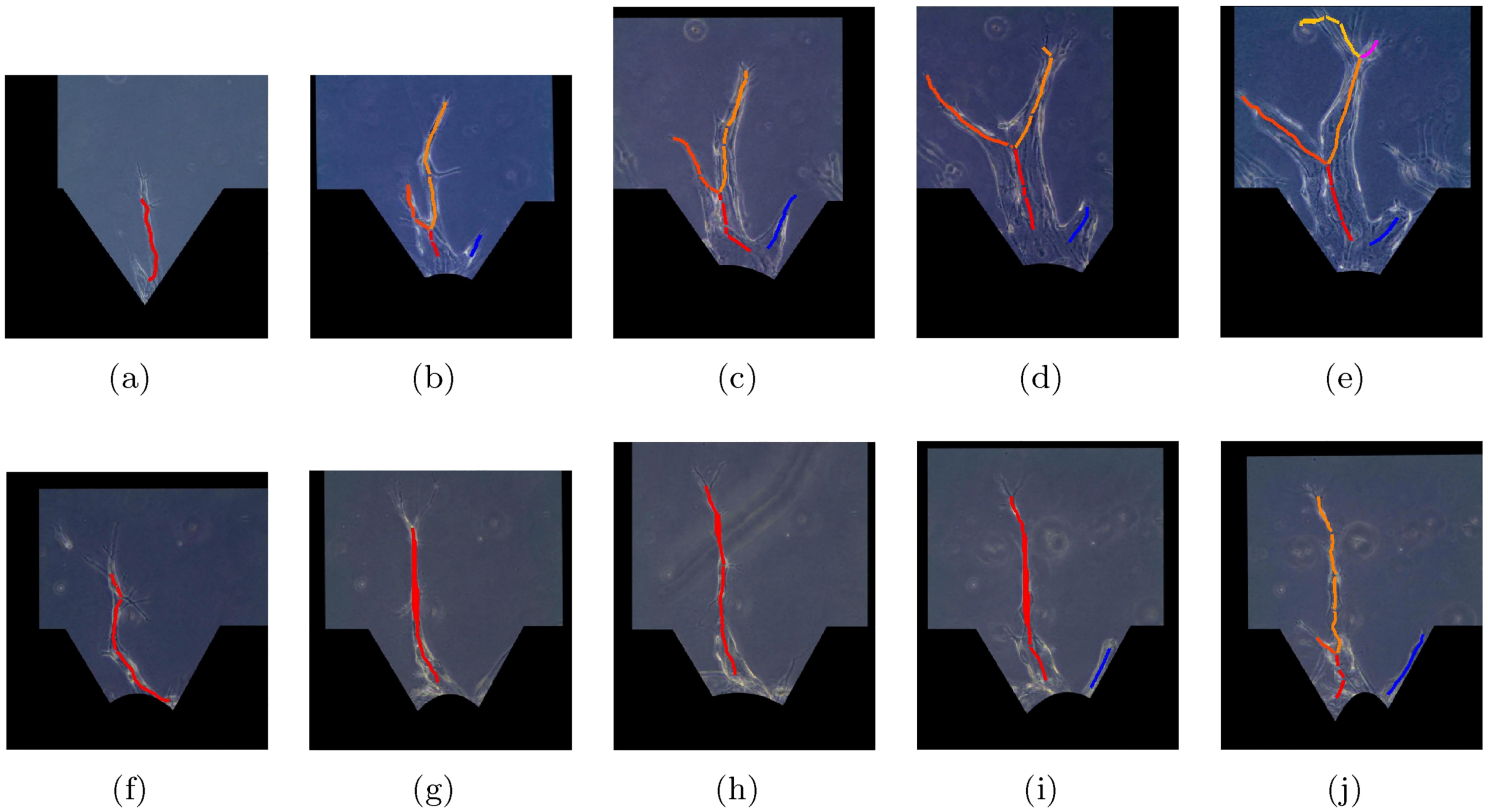
Examples for branch tracking of angiogenic vessels within a sprouting region from day 5 to day 9. (a) to (e) for example 1 and (f) to (j) for example 2.

Fig 13 illustrates results of branch formation tracking from two example datasets. The angiogenic vessels within a sprouting region are shown over different days (from day 5 to day 9). When new branch node appears, the newly formed branch is represented by a new centerline with a new color (indicated in Fig 13).

We tested the AVFTS on 20 sprouting regions, each of which contains 5 to 6 frames. In order to evaluate the performance of the AVFTS on branch tracking quantitatively, precision and recall measures are calculated. Precision is the fraction of the associated branches that identify the actual branch trajectories, and recall is the fraction of the actual branch trajectories that are identified. By comparing with the ground truth obtained from manual tracking by visual inspection, the precision is estimated as 97.3% and the recall is estimated as 93.9%.

Skeletonization is a key step in the AVFTS. Instead of a fixed segment threshold, we are exploring to adjust the segment threshold based on the branch information at later days, expecting to further improve the performance of the AVFTS.

### Application of the AVFTS

The proposed AVFTS system provides us numerical branch information including the length and width of each branch, and the number of branches for each sprouting region. By combining such branch information with the cell locations obtained in [26], cell phenotypes can be estimated. Biologists can also apply this proposed AVFTS system on the microscopy images obtained under different experimental conditions when they study the influence of different angiogenic factors on angiogenic vessel morphologies. As an illustration, we provide quantitative comparisons of the angiogenic vessels formed under different S1P conditions.

#### Cell phenotype identification

As explained, the dynamic competition of cell phenotypes serves an important function for angiogenic vessel formation [33]. Identification of tip and stalk cell phenotypes is important for investigating their different behaviors and unraveling the mechanisms for their inter-transition [59, 60].

Earlier studies have shown that tip cells are at the leading position in the branches in order to sense the stimuli and lead the way [1, 20, 61, 62]. Since we obtained the cell locations in [26] and branch information in this paper, the phenotype for each cell can be identified by considering its relative location in the branch.

**Fig 14:**
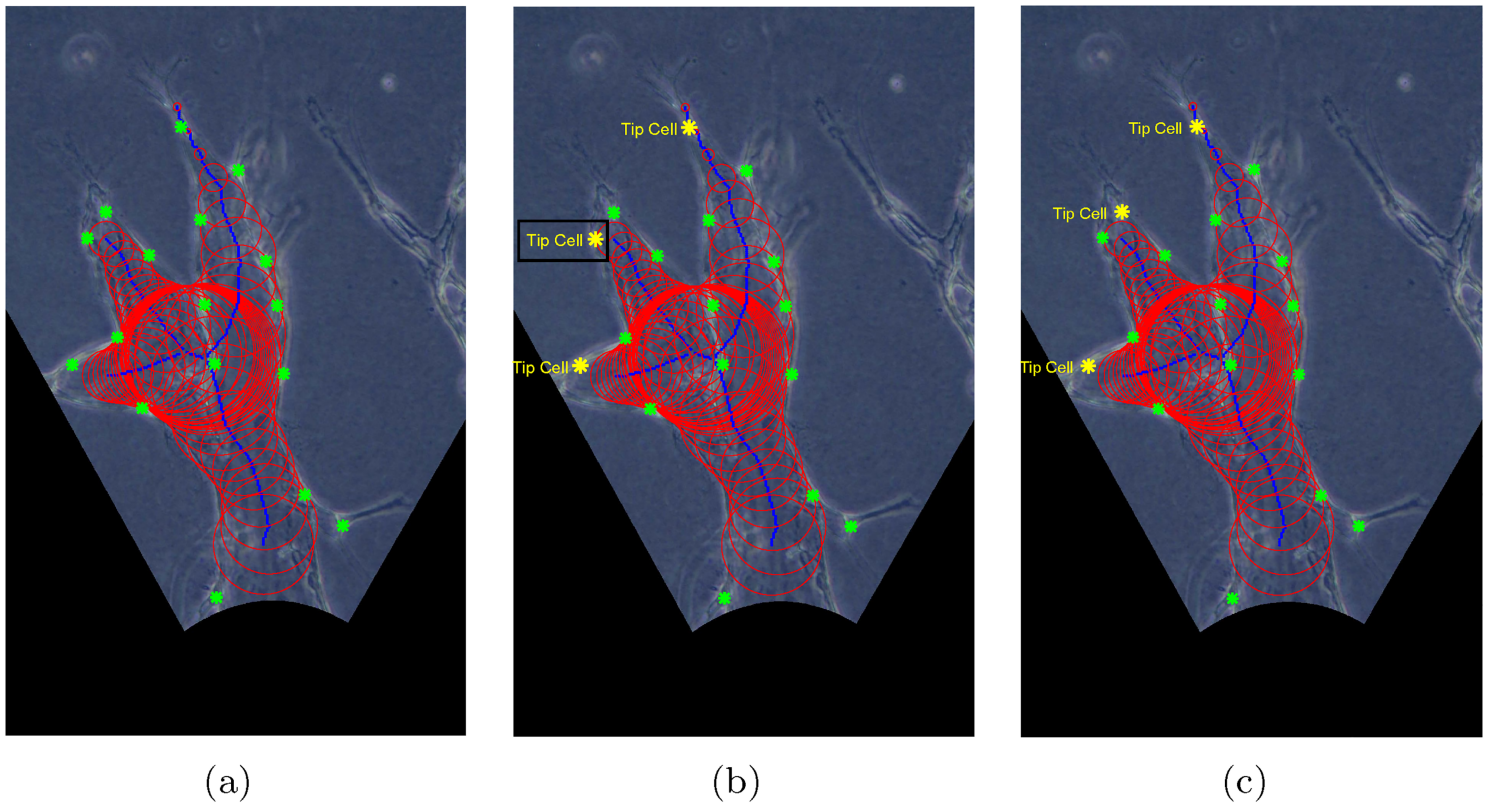
An example of tip cell identification result. (a) Angiogenic vessels with both cell and branch information: cell locations plotted as green stars, branch skeletons plotted as blue lines, and branch radii plotted as red circles. (b) Tip cells identified based on the distances between the cells and the end nodes only: tip cells plotted in yellow stars. (c) Tip cells identified with our approach: tip cells plotted in yellow stars.

In Fig 14(a), we provide an example of angiogenic vessels with both cell and branch information. The cell locations, branch skeleton, and the radii of the vessels are plotted as green stars, blue lines, and red circles respectively.

Each branch is headed by a tip cell [1]. In other words, the number of tip cells equals to the number of branches in the angiogenic vessels in a sprouting region. For instance, the angiogenic vessels in Fig 14(a) have 3 branches, and each of them has one tip cell.

As explained, each branch is indicated by the nodes it passes. For instance, branch 1 in Fig 9(d) is represented by [1 2 4 6], which consists of three segments [1 2], [2 4], and [4 6]. Since tip cells lead the way, they are expected to be located in the last skeleton segment which is farthest away from the monolayer, e.g. segment [4 6] for branch 1 in Fig 9(d). As a consequence, we can concentrate on the last skeleton segment in a branch to identify its corresponding tip cell.

**Fig 15:**
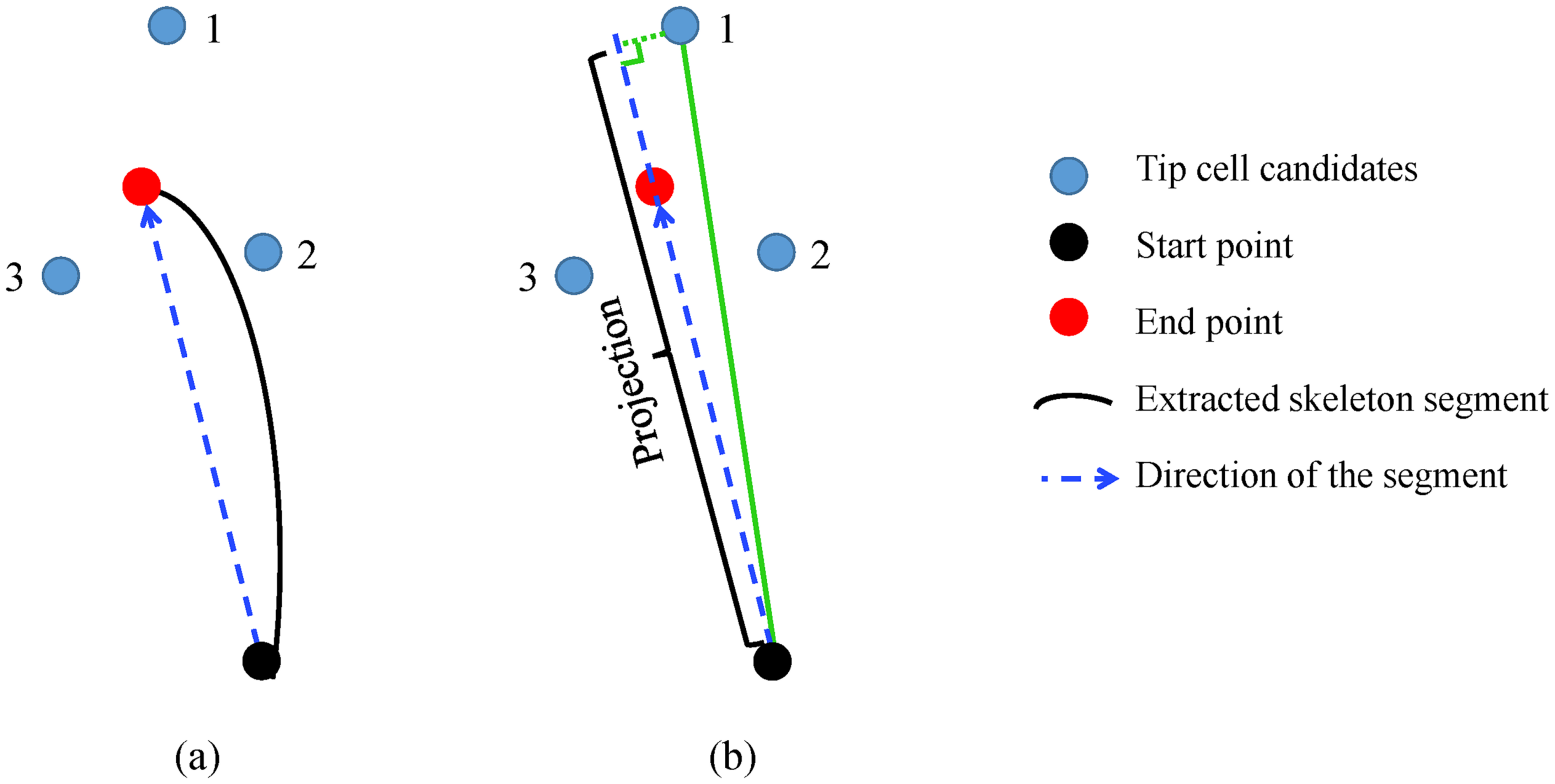
Diagram of tip cell identification. (a) Tip cell candidates. (b) Tip cell identification.

Fig 15(a) illustrates the diagram of the last segment in a branch with start point (black circle), end point (red circle), and the skeleton coordinates (black curved line). For each branch, our first step is to determine the tip cell candidates by selecting the *N*_tip_ cells closest to the end point of the last segment. Based on the observations, tip cell and its two neighboring stalk cells are the closest cells to the end point. Thus, *N*_tip_ = 3 is used in this paper.

The blue circles in Fig 15(a) indicate the three selected tip cell candidates. As the distances are about the same, we are unable to determine which cell is the tip cell based on the distances alone. Since the tip cell is always at the leading position in the branch, it can be identified as the cell that migrates the farthest along the direction of the segment. The projection of one tip cell candidate *i* on the segment direction (blue dotted line with arrow in Fig 15(b)) is estimated as:

$$\begin{array}{c} p_{i}=\frac{v_{se}\cdot v_{si}}{|v_{se}|}, \end{array}$$ (20)

where **v**_*se*_ is the segment direction and **v**_*si*_ is the vector between the start point and tip cell candidate *i*:

$$\begin{array}{c} v_{se}=x_{e}-x_{s}, \end{array}$$ (21)

$$\begin{array}{c} v_{si}=x_{i}-x_{s}, \end{array}$$ (22)

with **x**_*e*_, **x**_*s*_ and **x**_*i*_ as the coordinates for the end point, start point, and the *i*th tip cell candidate respectively. The candidate with largest projection value will be selected as the tip cell.

The cell closest to the end point can be either a tip cell or stalk cell. Therefore, selecting the cell nearest to the end point as the tip cell is not always a suitable choice. For instance, the cells depicted in Fig 14(b) by yellow stars are the tip cells identified based on the distances only. It is noteworthy that the cell within the black rectangular box is wrongly identified. By incorporating the projection estimated by Eq (20), this kind of errors can be avoided. The correctly identified tip cells are shown in Fig 14(c).

#### Investigation of S1P

As explained, the investigation of branching and anastomosis, which are key processes for the formation of a mature vascular, would lead to a better understanding of tip/stalk inter-transition. However, to our knowledge, the existing angiogenic studies in 3D *in vitro* experiments with HMVECs are unable to culture long vessels which are required for branching and anastomosis to occur.

S1P is involved in angiogenesis to stimulate cell invasion, lumen formation, and branching morphogenesis. Nguyen et al. have shown that S1P increases the average vessel length by increasing the migration of HUVECs [15]. Therefore, we employ S1P in our angiogenic experiments to form long angiogenic vessels. We consider three conditions of S1P (see Table 1).

**Fig 16:**
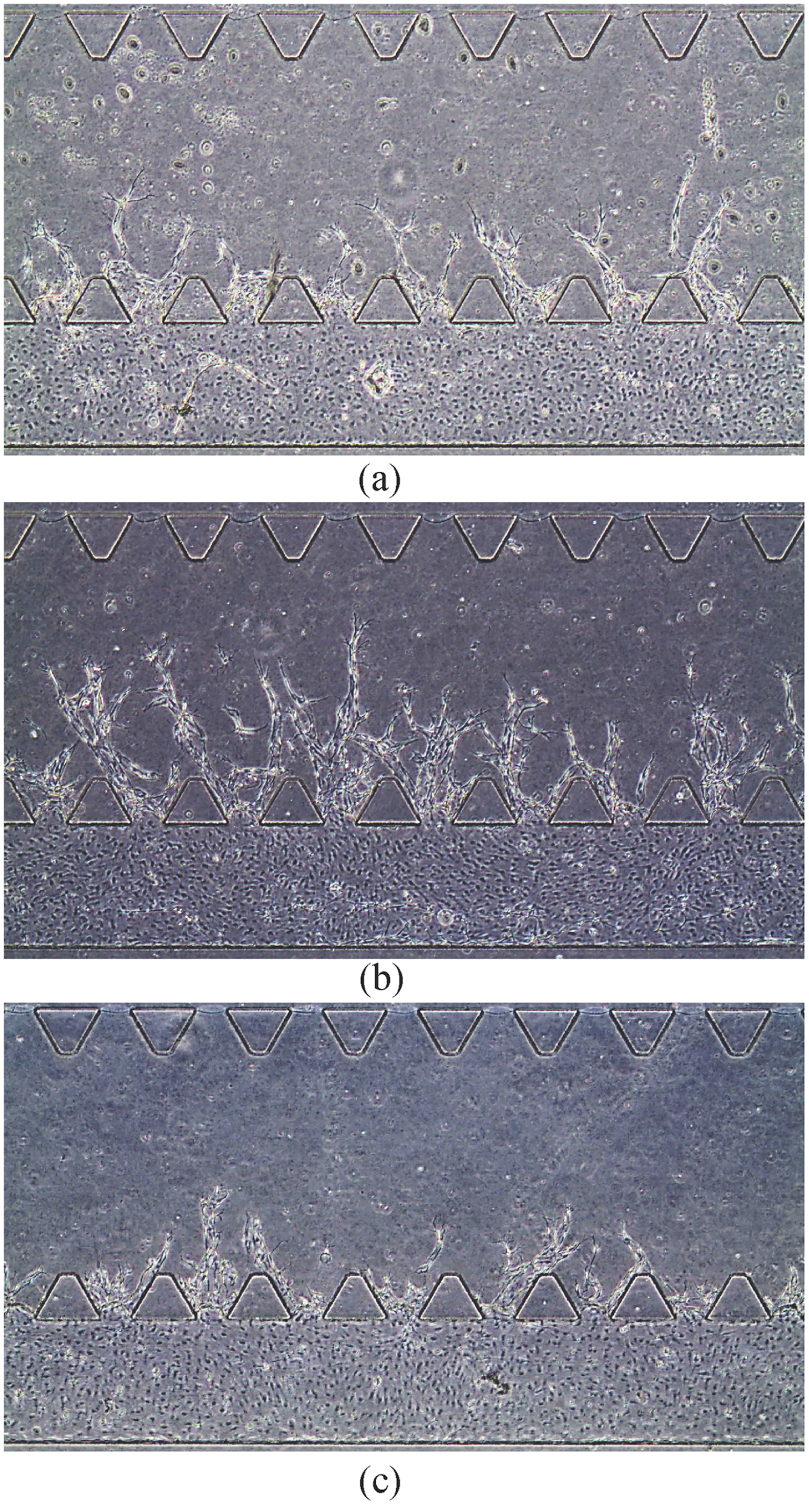
Angiogenic vessels observed by phase contrast microscopy (at 4x magnification) under different S1P conditions. (a) No S1P. (b) Positive S1P gradient. (c) Negative S1P gradient.

We observe the angiogenic vessels under different conditions by phase contrast microscopy at 4x and 20x magnification. The proposed AVFTS is applied to the phase contrast images at 20x magnification. To better illustrate the morphological difference of the angiogenic vessels under different S1P conditions, we provide their 4x phase contrast images (all acquired at day 9) in Fig 16. From the example in Fig 16, we can observe that the vessels from positive S1P gradient (see Fig 16(b)) are longer than in the other two conditions.

To quantitatively compare the vessel morphologies for the different S1P conditions, we apply the proposed AVFTS on the end-point phase contrast images obtained at 20x magnification. Numerical parameters including the branch length, branch radius, and the number of branches in each sprouting region can be estimated.

**Fig 17:**
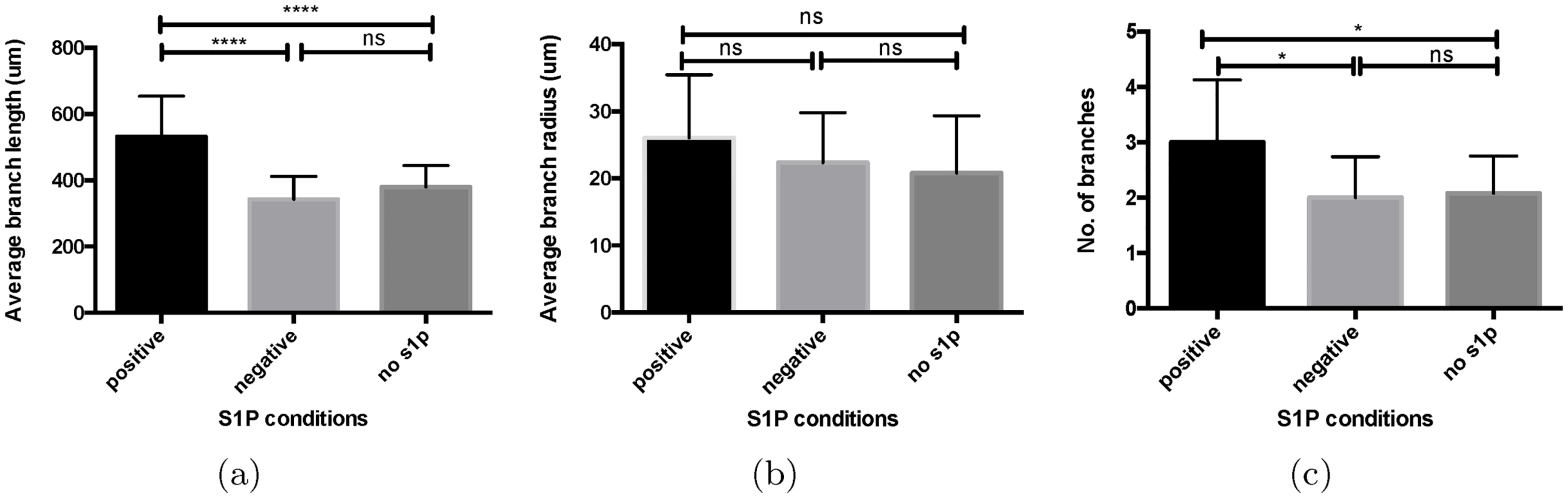
Quantitative comparison of vessel morphologies under different S1P conditions. (a) Branch length (*N* = 24 − 36). (b) Branch radius (*N* = 24 − 36). (c) The number of branches in a sprouting region (*N* = 12). The error bars give the mean and standard deviation.

We apply one-way analysis of variance (ANOVA) to examine the differences among the three S1P conditions on the branch length, branch radius, and the number of branches in a sprouting region. The P values for the ANOVA tests are shown in Fig 17. The symbols in Fig 17 are explained in Table 4.

**Table 4:** Symbols for different P values ranges.

P value	Symbol	Meaning
*P* > 0.05	ns	not significant
0.01 < *P* ≤ 0.05	*	moderately significant
0.001 < *P* ≤ 0.01	**	significant
0.0001 < *P* ≤ 0.001	***	very significant
*P* ≤ 0.0001	****	extremely significant

Fig 17(a) compares the average branch length for the different S1P conditions, showing that a positive S1P gradient induces significantly longer branches than the other two conditions. This observation implies that S1P in the medium channel regulates angiogenic sprouting by increasing ECs migration and vessel elongation, whereas S1P has no such effect in the cell channel.

The comparison of the average branch radius for the different S1P conditions is shown in Fig 17(b). It indicates that S1P does not lead to significant difference in the average vessel width among the three conditions.

As illustrated in Fig 17(c), the average number of branches in a sprouting region under the a positive S1P gradient is larger than the other two conditions. Branching happens when the sprout extends over some distance, and there is sufficient space locally for a new branch to form [8]. We hypothesize that a positive S1P gradient increases the vessel length, leading to higher possibility for branching to occur.

**Fig 18:**
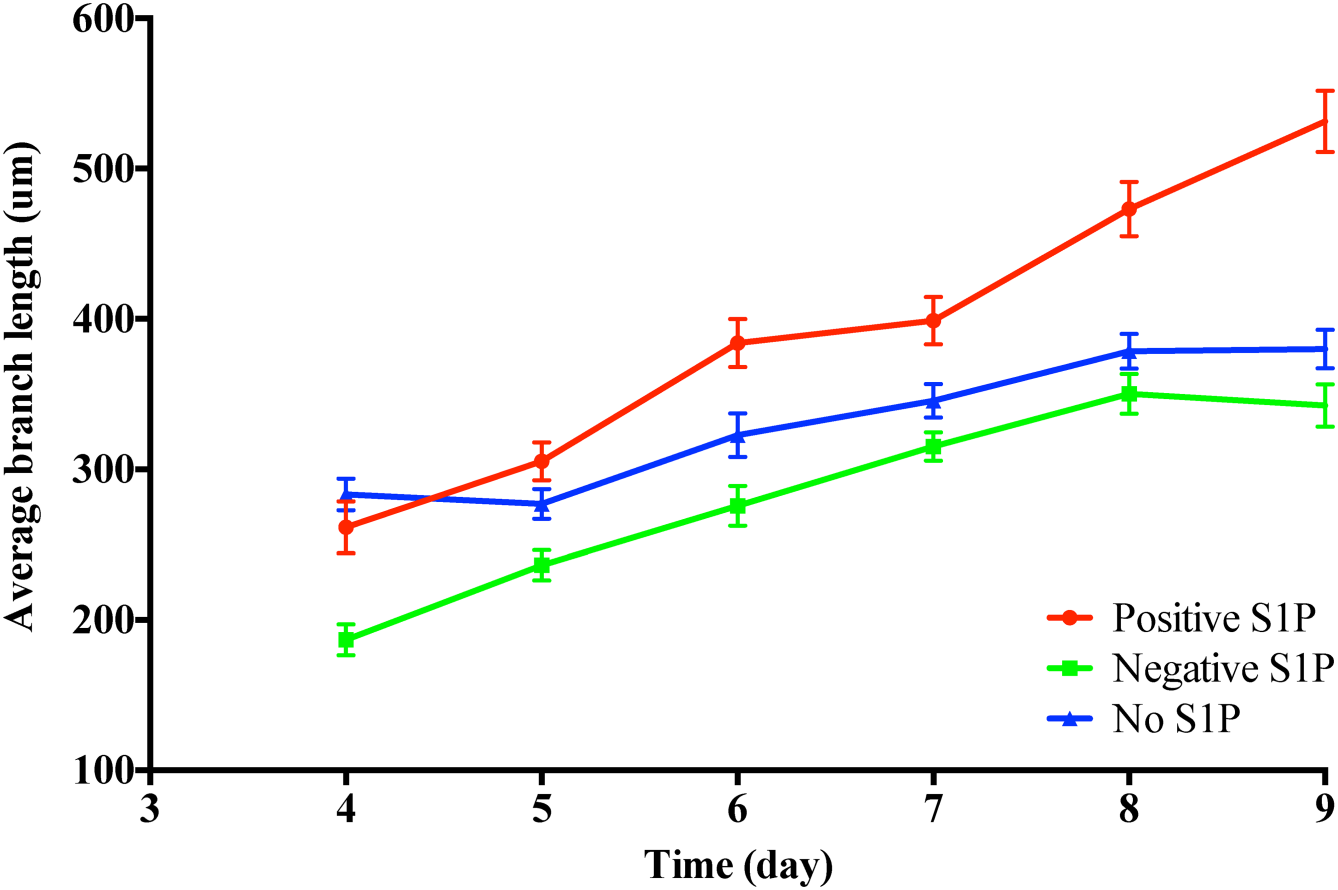
Change of average branch length over time for different S1P conditions. The error bars give the mean with SEM.

Fig 18 shows how the average branch length changes from day 4 to day 9 for different S1P conditions. We also plot the standard error of the mean (SEM) bars [63] to compare these three S1P conditions. The large gaps between bars between the positive S1P gradients and the other two conditions suggest that positive S1P gradients induce longer angiogenic vessels consistently over the 9-day period. More specifically, at day 8, the vessels keep extending in the angiogenic experiments with positive S1P gradients, whereas they almost stop growing for the other two conditions.

**Fig 19:**
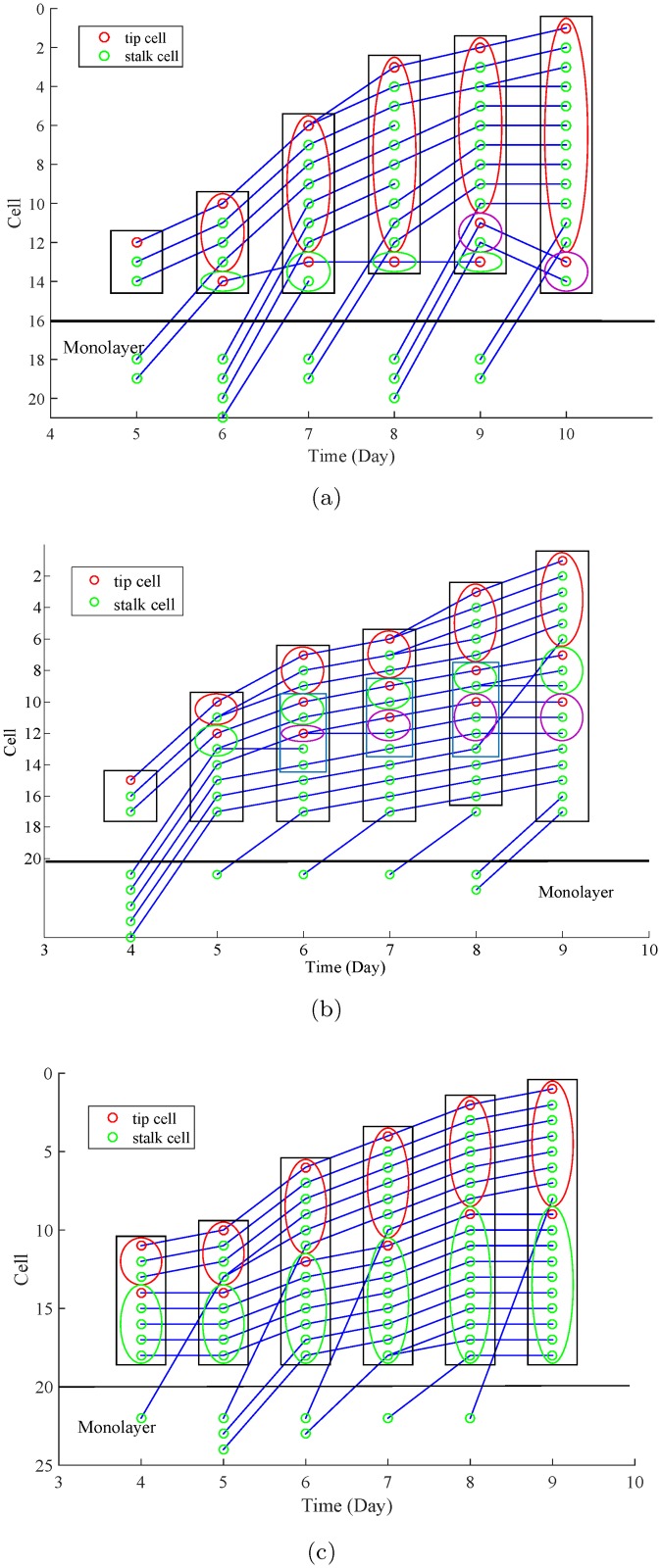
Examples of the novel cell lineage plots for the three different S1P conditions. (a) No S1P. (b) Positive S1P gradient. (c) Negative S1P gradient. Tip and stalk cells are plotted as red and green circles respectively. The cells within a same branch are included in a same ellipse. The ellipses with a same color represent a same branch over time. Cells shared by more than one branch are included in the rectangular but excluded from the ellipses. Cells migrating from the monolayer are plotted under the black horizontal line.

By combining the cell trajectory information obtained in [26] and the branch trajectory information from this paper, we generate a novel type of cell lineage plot, as shown in Fig 19. Compared with conventional cell lineage plots, such cell lineage plot not only provides cell migration and proliferation histories, but also demonstrates the cell phenotypic changes and branch information. For instance, tip and stalk cells are plotted as red and green circles respectively. The cells within a same branch are included in a same ellipse. The ellipses with a same color represent a same branch over time. For some of the cells, they locate at the skeletons which are shared by more than one branch. These cells are included in the rectangular but excluded from the ellipses. Cells migrating from the monolayer are plotted under the black horizontal line.

We provide examples of the novel cell lineage plots for no S1P, positive S1P gradient, negative S1P gradient in Fig 19(a), 19(b) and 19(c) respectively. We can see that:

- In positive S1P gradient (see Fig 19(b)), all the branches are originally from a same tip cell. A new branch is formed when a stalk cell transit to tip cell between day 5 and day 6.
- For the other two conditions in Fig 19(a) and 19(c), the branches are originally from different tip cells. New branches are formed when the cells in the monolayer are activated to become tip cells.

## Conclusion

In this paper, we have developed the AVFTS to automatically track the vessel formation, to extract quantitative vessel information, and to identify cell phenotypes from the experimental time-lapse phase contrast images. This system consists of preprocessing, skeletonization, and branch tracking. We first preprocess the experimental images to obtain the binary shapes. Next, we skeletonize the binary vessel shapes into segments by DT and AFMM. We then remove the specious segments that are not part of vessels by a binary SVM classifier. Lastly, we associate the branches over time through the Hungarian method. We are able to track the branch formation and extract branch information such as branch length, width, and number of branches in each sprouting region.

Based on the cell locations from [26] and branch information from AVFTS, we distinguish tip and stalk cells in a branch by considering both the distance of the cells to the end point of their last segment and the projection estimated by Eq (20). We also generate novel cell lineage plots by combining cell and branch information. These plots provide crucial information (e.g. the number of cells, cell proliferation histories, cell phenotypic changes, and branch information) to biologists who are interested in studying collective cell behaviors in angiogenesis.

Moreover, the proposed AVFTS enables biologists to investigate the influence of different angiogenic factors by extracting quantitative vessel information such as vessel length, vessel width, and number of branches from microscopy images under different experimental conditions. As an illustration, we applied AVFTS to data from the angiogenic experiments with different S1P conditions to extract and compare the morphological changes in the vessels over time. We observed that positive S1P gradients promote angiogenic vessel growth and lead to more branches in a sprouting region.

Existing computational models for angiogenesis rarely incorporate the occurrence of branching and anastomosis. In the future, we may develop computational models from the numerical data (e.g. cell locations, cell phenotypes, and branch information) generated by the proposed systems. Such models might be able to predict the change of cell states (phenotypes) and cell coordinates (migration velocities). Branching and anastomosis may also be predicted, since these are processes often accompanied with cell phenotypic changes.
